# Dysregulation of MiRNAs in schizophrenia in an Egyptian patient population

**DOI:** 10.1038/s41598-025-01831-4

**Published:** 2025-05-16

**Authors:** Nabila M. Adly, Dalia Khalifa, Shaimaa Abdel-Ghany, Hussein Sabit

**Affiliations:** 1 Department of Medical Biotechnology, College of Biotechnology, Misr University for Science and Technology, P. O. Box 77, Giza, Egypt; 2 Psychiatry Department, Kasr Al Ainy Hospitals, Cairo University, Giza, Egypt; 3 Department of Environmental Biotechnology, College of Biotechnology, Misr University for Science and Technology, P. O. Box 77, Giza, Egypt

**Keywords:** Schizophrenia, MiRNA dysregulation, Cognitive function, Biomarkers, Personalized medicine, Molecular medicine, Laboratory techniques and procedures

## Abstract

Schizophrenia (SZ) is a complex neuropsychiatric disorder influenced by genetic, environmental, and epigenetic factors, including miRNA dysregulation. This study explored the diagnostic and therapeutic potential of miRNAs in SZ, focusing on seven key miRNAs: miR-137-3p, miR-34a-5p, miR-432-5p, miR-130b-3p, miR-346, miR-195-5p, and miR-103a-3p. Results revealed significant dysregulation of miR-137-3p, miR-195-5p, miR-346, and miR-103a-3p, highlighting their relevance to SZ pathology. Upregulation of miR-137-3p correlated with enhanced cognitive performance, as evidenced by improved scores on the Wisconsin Card Sorting Test (WCST) and Trail Making Test B (TMT-B). Conversely, miR-195-5p and miR-346 were strongly associated with cognitive processing speed, while miR-103a-3p downregulation was linked to reduced conceptual flexibility. Cluster analyses demonstrated that miRNA expression levels varied significantly based on antipsychotic treatment and receptor targeting, suggesting potential regulatory effects of medication. Importantly, miRNAs were measured in PBMCs, highlighting their feasibility as non-invasive biomarkers. The study underscores the diagnostic value of miRNAs, offering a promising avenue for early detection and personalized interventions in SZ. Future research should validate these findings across diverse cohorts and investigate miRNA-based therapeutic strategies. By integrating miRNA profiling into clinical practice, this study provides a foundation for advancing precision medicine in SZ management.

## Introduction

Schizophrenia is a chronic brain disease affecting approximately 1% of the world population that causes a severe health burden^1^. It usually occurs early in adolescence or adulthood, persists for most of an individual’s life, and is recognized as one of the top 15 leading causes of disability worldwide^2,3^.

The worldwide prevalence of schizophrenia as a neurodevelopmental disorder is up to 1.5% with apparent gender differences (e.g., lower incidence and different temporal patterns in women)^4^. It was found that there is an increased risk of violent crime among patients diagnosed with schizophrenic disorders at 80.6%, followed by bipolar disorder at 7.3% and mental retardation at 8.1%. Most of the crimes committed were homicide (56.4%) and physical assault (20.6%)^5^. Schizophrenia is characterized by negative symptoms, which include affective flattening, alogia, anhedonia, asociality, and avolition. Paranoid delusions, hallucinations, unusual behavior, and positive formal thought disorder are positive signs. Poor cognitive symptoms include difficulty focusing, executive functioning problems, and decreased working memory^6^.

Research has found various schizophrenia risk factors. The disease is caused by biopsychosocial factors like genetic, environmental, epigenetic, neuroanatomic, neurochemical, and biological abnormalities. Stress, cannabis use, pregnancy, and birth difficulties may all increase schizophrenia risk. Based on genetic studies, schizophrenia is a polygenic syndrome with hundreds or thousands of genetic loci. Genome-wide association studies discovered over 100 genetic sites with common alleles of varied effects^7–10^.

Despite genes’ major influence on schizophrenia onset, environmental variables may also be important. Environmental risk factors for schizophrenia include prenatal and perinatal complications, cannabis use, childhood trauma, social stressors, and malnutrition^11^, social stressors^12^. Only obstetric problems, stressful events, childhood adverse events, cannabis usage, and serum folate levels have been linked to schizophrenia^13,14^.

The diagnosis of schizophrenia is primarily clinical, relying on established criteria such as the Diagnostic and Statistical Manual of Mental Disorders (DSM-5) or the International Classification of Diseases (ICD-10). A diagnosis requires the presence of two or more core symptoms as delusions, hallucinations, disorganized speech, disorganized or catatonic behavior, and negative symptoms for at least six months, with one month of active-phase symptoms^15^. Crucially, at least one of these symptoms must be delusions, hallucinations, or disorganized speech. However, the heterogeneity of the disorder and symptom overlap with other psychiatric conditions often make diagnosing schizophrenia a challenging process, requiring comprehensive psychiatric evaluations, detailed patient histories, and neuropsychological testing.

Recent research has shifted toward identifying biological markers to complement clinical diagnostic criteria. Neuroimaging studies have revealed structural abnormalities in schizophrenia, such as enlarged ventricles and reduced gray matter volume, particularly in the prefrontal cortex and hippocampus—regions critical for cognitive function and emotional regulation. At the neurochemical level, dysfunctions in dopamine, glutamate, and gamma-aminobutyric acid (GABA) neurotransmission have been strongly implicated in the pathophysiology of schizophrenia^16^.

The miRNAs are short, single-stranded, non-coding RNA molecules that consist of 18–22 nucleotides that have been widely identified in eukaryotic genomes and play critical parts in the occurrence and development of many diseases^17–19^.

MiRNAs that regulate neuronal development are neuron-specific or highly produced in the brain. This includes regulating neural progenitor cell proliferation, neuronal fate, circuitry establishment, synaptic plasticity, and brain health^20–22^. Therefore, it is imperative to maintain a well-functioning miRNA biogenesis process to develop a healthy brain properly. Dysregulation of genes involved in encoding miRNAs or components of the miRNA biogenesis machinery has been linked to psychiatric disorders, including schizophrenia^23,24^.

The growing recognition of the importance of miRNA in regulating gene expression during neurodevelopment has led to an increasing focus on whether the disruption of miRNA regulation could underline CNS disorders that are characterized by complex changes in CNS-related gene expression, such as schizophrenia. Studies in developing rodents show that miRNAs display complex spatial and temporal patterns of expression^25,26^. Amongst the first studies to explore miRNA expression in subjects with schizophrenia, Perkins et al. reported lower levels of the miRNA miR-30B in the prefrontal cortex of subjects with schizophrenia^27^. Several subsequent studies have examined changes in miRNA expression in the postmortem CNS of subjects with schizophrenia^28–31^. This suggests that schizophrenia involves the disruption of a substantial proportion of the brain’s miRNA-mediated transcriptional regulation^30–32^.

GWAS studies verify that rs1625579, located within miR-137 genes (1p21.3), was significantly associated with schizophrenia. The target genes regulated by miR-137 include CUB and Sushi multiple domains 1 (CSMD1), WW domain binding protein 1-like (C10orf26), calcium voltage-gated channel subunit alpha1 C (CACNA1C), transcription factor 4 (TCF4), and zinc finger protein 804 A (ZNF804A). These genes have been identified as genetic risk factors for schizophrenia^33^ and are part of the glucocorticoid receptor-dependent signal transduction network^34^. Furthermore, this miRNA has been identified as regulating genes that are important for the GABA system^35^.

Functionally, most reports suggest the rs1625579 genotype impacts cognitive function. In patients with schizophrenia, the T/T genotype is associated with working memory deficits, with patients showing poor performance on the brief assessment of cognition in schizophrenia (BACS). These subjects also show worse negative symptoms on the positive and negative syndrome scale (PANSS). Even amongst studies that fail to find an association between MIR137 SNPs and the incidence of schizophrenia, some studies have shown a correlation between the rs1625579 genotype and cognitive performance^36–38^.

Amongst the first studies to explore miRNA expression in subjects with schizophrenia, Perkins et al. reported lower levels of the miRNA miR-30B in the prefrontal cortex of subjects with schizophrenia^27^. The MIR130B gene is located within cytogenetic band 22q11.21. This region is highly implicated in schizophrenia both as the location of several schizophrenia candidate genes, including catechol-O-methyltransferase (COMT), and due to its involvement in DiGeorge syndrome. Individuals with DiGeorge syndrome carry deletions spanning chromosomal band 22q11.2 and have an increased risk of developing a psychotic illness, with a quarter of suffers meeting the criteria for schizophrenia under the Diagnostic and Statistical Manual of Mental Disorders^36^.

Studies indicated that miR-195 belongs to the miR-15/16/195/424/497 miRNA family, which is closely related to cell propagation and apoptosis^39^. It is abundantly produced in the brain tissue and peripheral blood and has been shown to target the expression of the brain-derived growth factor (BDNF) gene^40,41^. This miRNA binds directly to the 3′-untranslated region (UTR) of the BDNF mRNA and inhibits BDNF protein translation. BDNF regulates neuroplasticity, inhibits the apoptosis cascade, and increases the levels of several cellular proteins required for neurogenesis, neuronal proliferation, and survival^42,43^. The gene encoding miR-346 was located at the intron glutamate receptors ionic δ1 gene (GRID1), and the GRID1 gene is the susceptibility gene for schizophrenia^44^. Numerous studies have indicated that reduced BDNF levels are related to impaired cognitive function in individuals with schizophrenia^45^. MiR-30a-5p and miR-195 may regulate the gene expression of brain-derived neurotrophic factors (BDNF), so it is suggested that miR-195 may be involved in the pathogenesis of schizophrenia by regulating BDNF^46^.

Overall, hsa-miR-103a-3p, hsa-miR-137-3p, hsa-miR-130b-3p, hsa-miR-34a-5p, hsa-miR-432-5p, hsa-miR-195-5p, and hsa-miR-346 have been reported to play a role in the onset and development of schizophrenia^47–51^.

The present study aims to evaluate the role of hsa-miR-103a-3p, hsa-miR-137-3p, hsa-miR-130b-3p, hsa-miR-34a-5p, hsa-miR-432-5p, hsa-miR-195-5p, and hsa-miR-346 in schizophrenia patients compared to healthy individuals in a sample of Egyptian patients.

## Subjects, material, and methods

### Study design

This case-control study was conducted at the College of Biotechnology, Misr University for Science and Technology, Giza, Egypt, and the Psychiatric Department of Kasr Al Ainy Hospital, Cairo, Egypt.

### Study sample

This study was conducted from October 2023 to December 2023. Blood samples were randomly collected from 40 male patients with schizophrenia who were attending the psychiatric unit at Kasr Al Ainy Hospital. The age range of the participants was 18 to 45 years. The clinical diagnosis of patients was confirmed by psychiatrists based on the criteria of the Diagnostic and Statistical Manual of Mental Disorders, Fifth Edition (DSM-5)^52,53^.

### Inclusion criteria

The study included two groups. The schizophrenia group comprised individuals aged 18 to 45 years who were diagnosed with schizophrenia based on DSM-5 criteria, had no significant medical history, demonstrated clinically average intelligence, had no history of substance abuse, and had not undergone electroconvulsive therapy (ECT) within the past three months. The control group consisted of individuals aged 18 to 45 years with no history of psychiatric or neurological disorders, no significant medical conditions, clinically average intelligence, no family history of psychotic disorders, and no history of substance abuse. All participants were screened through a comprehensive clinical evaluation to confirm adherence to these criteria and to minimize potential confounding factors.

#### Exclusion criteria for both groups

To ensure the validity and reliability of the results, individuals with a past or current history of drug or substance abuse, neurological disorders, hematological disorders, clinically below-average intelligence (intellectual disability), or a history of traumatic brain injury were excluded from the study. Substance abuse was a key exclusion criterion, as chronic drug use can induce neurochemical and cognitive alterations that may obscure or confound schizophrenia-specific abnormalities, making it challenging to delineate the disorder’s true effects. Similarly, individuals with neurological disorders, including epilepsy, stroke, or neurodegenerative conditions, were excluded due to their potential impact on cognitive function, brain structure, and gene expression, which could lead to misinterpretation of schizophrenia-associated findings. Hematological disorders were also excluded, as variations in blood composition, such as anemia or coagulation disorders, could influence peripheral blood mononuclear cell (PBMC) composition and miRNA expression levels, thereby introducing confounding variables. Additionally, individuals with intellectual disability (low IQ) were not included, as the neuropsychological assessments employed in this study require an average IQ for valid and reliable evaluation. The inclusion of participants with intellectual disabilities could have biased the interpretation of schizophrenia-related cognitive impairments. These exclusion criteria were rigorously applied to minimize confounding factors and ensure that the observed differences in miRNA expression and cognitive function were specifically attributable to schizophrenia.

### Sample size calculation

The sample size was calculated using G Power, Version 3.1.9.4^54^. With a significance level of α = 0.05 and a power of 0.80. Based on the effect size, the minimum required sample size for a t-test comparing two independent groups was determined to be *N* = 54, with an allocation ratio of 3:1 (schizophrenia group: control group). This resulted in a sample distribution of 40 participants in the schizophrenia group and 14 in the control group.

### Ethical consideration

Written informed consent was obtained from all the participants after they were informed about the nature and goals of the study. The study protocol was approved by Cairo University’s IRB (N-344-2023). All procedures were in accordance with the Declaration of Helsinki.

## Materials

### Chemicals and kits

The chemicals and kits used in this study were the miRCURY LNA miRNA PCR Starter Kit Human, the miRNeasy Mini Kit, the MiRCURY LNA SYBR Green PCR Kit, QIAzol^®^ Lysis Reagent, and RNase-free water, all manufactured by QIAGEN, Germany. Additional materials included the Secoll™ Lymphocyte Separation Solution from Serana, Germany, and chloroform and ethanol from Piochem, Egypt.

### Studied MiRNAs

The miRNAs used in this study were hsa-miR-103a-3p, hsa-miR-137-3p, hsa-miR-130b-3p, hsa-miR-34a-5p, hsa-miR-432-5p, hsa-miR-195-5p, and hsa-miR-346. All are sourced as miRCURY LNA miRNA PCR Assays from QIAGEN, Germany.

### Research strategy

#### Trail making test B (TMT-B)

TMT-B is a commonly used cognitive assessment tool used to measure cognitive performance and identify potential impairments^55^. The TMT-B assesses working memory, mental flexibility, visual scanning, and set-shifting ability. The test began with an explanation of its purpose to the participant. A sheet displaying 25 circles with numbers (1 to 13) and letters (A to M) was then provided. The participant was instructed to start at 1, draw a line to A, and then proceed by alternating between numbers and letters in ascending order (e.g., 1-A-2-B-3-C), continuing this sequence until all circles were connected. Any mistakes made during the test were promptly pointed out and corrected. Although the test typically takes 3 to 4 min to complete, it was set to stop after 5 min if unfinished.

#### Wechsler adult intelligence scale III (WAIS-III)

It has been developed to assess various cognitive domains, including verbal comprehension, perceptual reasoning, working memory, and processing speed^56^.

#### Similarities

It is a part of the verbal comprehension scale, which measures abstract reasoning and the ability to identify commonalities between words or concepts. Impairments in this area may indicate difficulties in abstract thinking. The purpose of the test was explained to the participant. Pairs of words or concepts were then presented to them one at a time, and they were asked to describe how these items are alike or what common features they share. Participants were encouraged to provide brief and concise responses.

#### Digit span

This test is a part of the Working Memory Scale, which assesses short-term memory and attention. It involves repeating a sequence of digits forward and backward, and Impairments may indicate working memory deficits. The purpose of the test was explained to the participant. A series of digits was then presented to them, one at a time, and the participant was asked to repeat the digits in the exact order they were given. As the test continued, longer sequences of digits were presented, and the participant continued to recall them accordingly.

#### Digit symbol coding

It is part of the Processing Speed Scale, which evaluates processing speed, visual-motor coordination, and attention. Impairments may suggest difficulties in information processing and psychomotor speed. The purpose of the test was explained to the participant, and a sheet containing number-symbol pairs was presented. The participant was instructed to look at the top of the page, where each symbol is paired with a number, and to write down the corresponding symbol for each number as quickly as possible. They were to work through the list sequentially (e.g., 1-A, 2-B, 3-C). Both accuracy and speed were emphasized, and the participant’s score was determined by the number of correct symbol substitutions completed within a 90-second time frame.

#### Positive and negative syndrome scale (PANSS)

The Positive and Negative Syndrome Scale (PANSS) is a clinical tool used to measure the symptom severity of individuals with schizophrenia. It assesses a range of positive symptoms, negative symptoms, and general psychopathology, which can include cognitive functions. A clinical interview was conducted, during which the patient was observed to assess various symptoms. Based on the interview, the patient was rated on a scale of 1 to 7 across 30 symptoms. The scale ranged from “1,” indicating the absence of symptoms, to “7,” indicating extreme symptoms.

#### General health Questionnaire-12 (GHQ-12)

The General Health Questionnaire-12 (GHQ-12) is a screening tool designed to identify short-term changes in mental health and assess levels of psychological distress. The purpose of this assessment was to detect potential psychiatric disorders, measure general psychological well-being, and screen for general (non-psychotic) mental health problems. The procedure involved conducting an interview, during which each question was read to the participants, and their responses were recorded. Each item on the GHQ-12 was scored from 0 to 3, with scores arranged by severity from left to right.

#### Wisconsin card sorting test (WCST)

The Wisconsin Card Sorting Test (WCST) is a neuropsychological test that assesses executive functions, such as set-shifting, flexibility, working memory, and abstract thinking. PEBL software involves a series of steps to ensure accurate assessment and scoring^57^. PEBL’s “Berg Card-Sorting Test-64” (PBCST-64), a free implementation of the Wisconsin Card Sort-64 (a shortened standardized form of WCST)^58^.

## Methods

### Blood sample collection

Peripheral blood (5 mL) was collected in EDTA-coated tubes from 40 schizophrenic patients attending the psychiatry unit at Kasr Al Ainy Hospital (age range: 18–45 years) and from 14 healthy control individuals. Control participants were recruited from Kasr Al Ainy Hospital staff, ensuring they had a comparable educational background to the schizophrenia group. They were screened to confirm the absence of psychiatric or neurological disorders. Peripheral blood mononuclear cells (PBMCs) were then isolated from these blood samples and subsequently stored at -18 °C until miRNA extraction was performed.

### MiRNA expression profiling

The expression levels of circulating miRNAs (miR-137-3p, miR-34a-5p, miR-432-5p, miR-130b-3p, miR-346, miR-195-5p, miR-103a-3p) were determined by two-step reverse transcription polymerase chain reaction (RT-PCR).

### Quantitative Real-time PCR

miRNA was extracted from EDTA-coated peripheral blood mononuclear cells samples of patients and controls using the miRNeasy Mini Kit (cat. no. 217004) following the manufacturer’s protocol. The extracted miRNA was converted into cDNA using the MiRCURY LNA miRNA PCR Starter Kit (cat. no. 339320) and reverse transcription primers. RNA samples were diluted to 5 ng/µL, and reverse transcription reactions were conducted in a thermal cycler, with incubation at 42 °C for 60 min and enzyme inactivation at 95 °C for 5 min. Quantitative real-time PCR (qPCR) was performed using the MiRCURY LNA SYBR Green PCR Kit (cat. no. 339345) and specific primers for each miRNA. The cDNA template was prepared by diluting reverse-transcribed cDNA, and qPCR reactions were carried out with an initial activation at 95° C for 2 min, followed by 40 cycles of denaturation at 95° C for 10 s and annealing/extension at 56° C for 60 s. Fluorescence data were collected in each cycle, and melting curve analysis was conducted from 60–95° C. Data analysis was performed using Global Mean Normalization (GMN), where the CT values of target miRNAs were normalized to the average CT of all detected miRNAs in each sample. Fold changes in miRNA expression were then calculated relative to healthy controls using the ∆∆CT method.

### Statistical analysis

The collected data was recorded and then presented and statistically analyzed using Statistical Package for the Social Sciences (SPSS) 25.0 for Windows (SPSS Inc., Chicago, IL, USA). Data processing involved editing and coding, followed by data entry on the computer. The data were then summarized and presented in tables and graphs. Qualitative data were summarized by number and percentage, while quantitative data were analyzed for normality using the Kolmogorov-Smirnov test, with normality assumed at *p* > 0.05. For statistical analysis, the Mann-Whitney test was used for nonparametric comparisons between two groups, while the Student’s t-test was applied for parametric data. Inter-group comparisons of categorical data were conducted using the chi-square test (X²) and Fisher’s Exact test. Multivariate logistic regression analysis to predict schizophrenia cases. All tests were two-sided, with significance set at *p* < 0.05, and *p* ≤ 0.001 considered highly significant. The Benjamini-Hochberg (BH) adjustment was applied to control the false discovery rate (FDR) and reduce the likelihood of false-positive results.

## Results

### Socio-demographic data

The socio-demographic data for the various participant groups was categorized into two groups for the study. Group A consisted of 40 individuals diagnosed with schizophrenia, with an average age of 32.55 ± 8.80. Group B comprised 14 healthy controls, with an average age of 30.35 ± 8.98. Notably, all participants were male and reported no history of substance abuse, as shown in Table (1).

**Table 1:** Differences between the studied groups according to their socio-demographic characteristics and smoking history.

Variable		Cases		Control		Chi-Square	*p*-value
		N.	%	N.	%		
Age		Mean ± SD = 32.55 ± 8.80	Range = 18.00–45.00	Mean ± SD = 30.35 ± 8.98	Range = 18.00–44.00		
	≤ 30	19	47.5%	7	50.0%	0.026	0.87
	˃30	21	52.5%	7	50.0%		
Education	Primary school	9	22.5%	1	7.1%	FET= 4.736	0.32
	Preparatory school	3	7.5%	0.0	0.0%		
	Secondary school	1	2.5%	2	14.3%		
	Institute of higher education	4	10.0%	2	14.3%		
	Diploma	18	45.0%	7	50.0%		
	Bachelor’s degree	5	12.5%	2	14.3%		
Smoking	Smoker	34	85.0%	6	42.9%	9.59	**0.002 (HS)**
	Non-smoker	6	15.0%	8	57.1%		

HS: Highly significant p˂0.01; FET = Fisher’s Exact test.

#### Disease onset

**Fig. 1:**
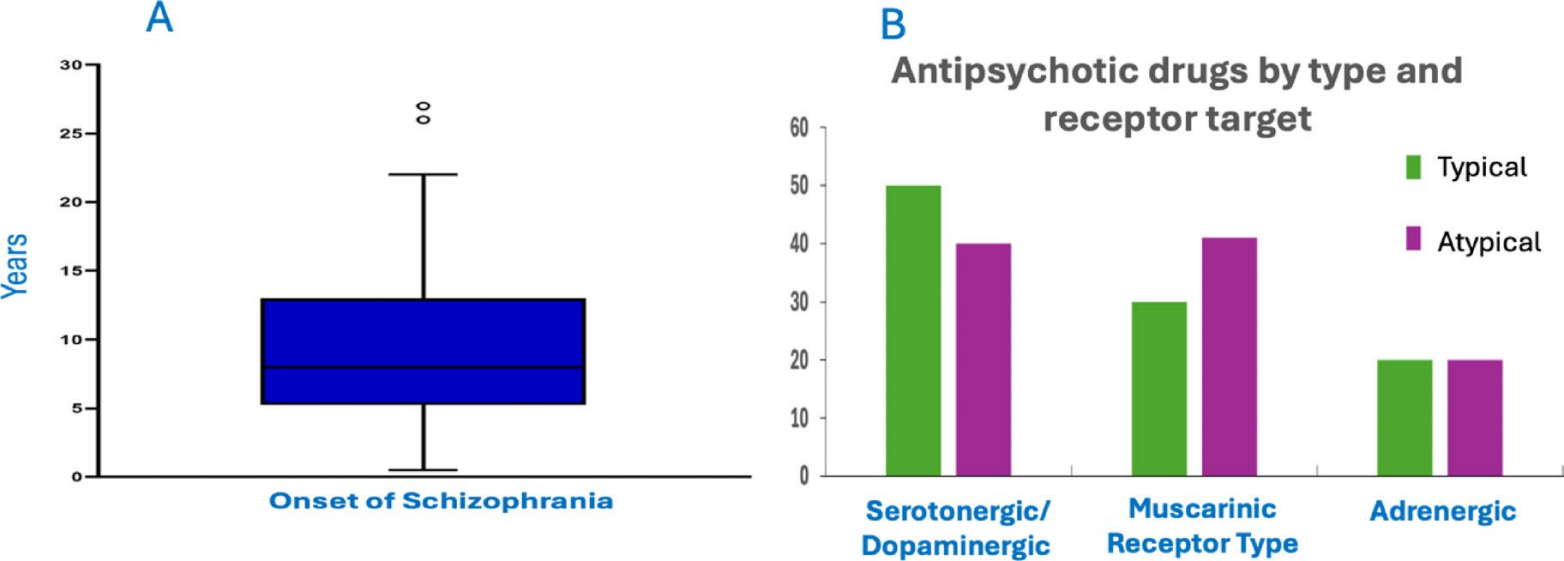
**A**: Box plot illustrating the distribution of the onset age of schizophrenia (in years). The median onset age is indicated within the interquartile range, while outliers reflect instances of later onset. **B**: Distribution of antipsychotic drugs prescribed for individuals with schizophrenia, categorized by drug type (Typical or Atypical) and receptor target (Serotonergic/Dopaminergic, Muscarinic, or Adrenergic). The graph highlights the preference for Serotonergic/Dopaminergic receptor targeting, particularly among typical antipsychotics, while atypical drugs exhibit a more balanced receptor target profile across Muscarinic and Adrenergic pathways.

The onset of schizophrenia typically occurs at younger ages, as indicated by the median onset age lying near the lower quartile, with moderate variability observed within the interquartile range. The whiskers extend from near zero to a maximum age of approximately 20, while two outliers above 25 represent cases with a later onset, underscoring the predominance of early-onset cases (Fig. 1A). Antipsychotic medications prescribed for individuals with schizophrenia are classified into two main categories: Typical (first-generation) and Atypical (second-generation) antipsychotics. These medications exert their effects by targeting various neurotransmitter receptors, primarily dopaminergic and serotonergic systems, as well as muscarinic and adrenergic receptors. The classification is based on both their mechanism of action and receptor-binding affinities, which influence their therapeutic effects and side-effect profiles. (Fig. 1B). This classification highlights the receptor-specific actions of these drugs and provides valuable insight into the distribution of treatment approaches within the studied population.

#### MiRNA analysis

**Fig. 2:**
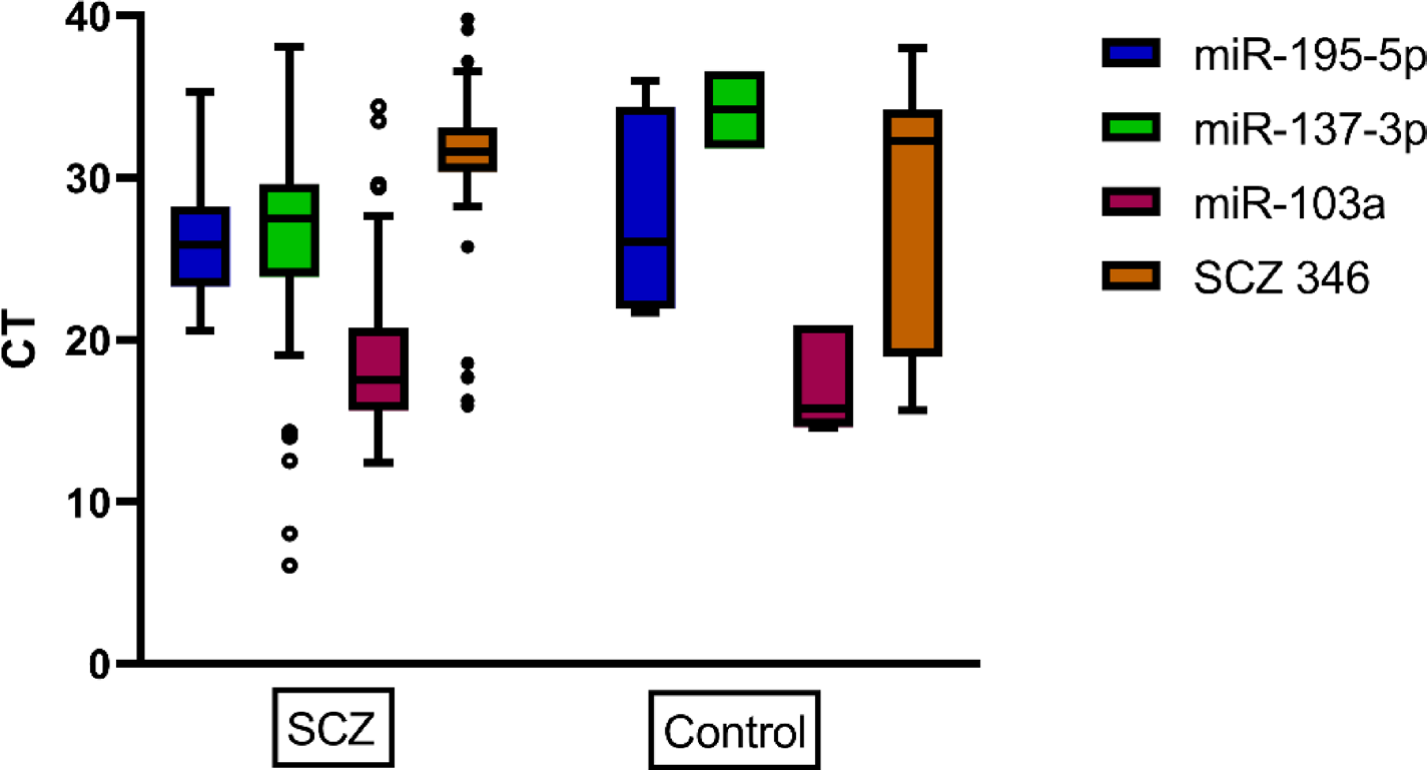
Comparison of CT values for selected miRNAs between schizophrenia (SCZ) cases and control groups. The boxplot shows significant differences in expression for miR-195-5p, miR-137-3p, and miR-103a-3p, miR-346, with lower CT values in the SCZ group for miR-137-3p and miR-195-5p, indicating higher expression levels. Statistical analysis revealed that miR-137-3p (*p* = 0.000), miR-103a-3p (*p* = 0.000), were highly significant (HS), while mi-R-346 (*p* = 0.01) and miR-195-5p were significant (*p* = 0.04).

**Fig. 3:**
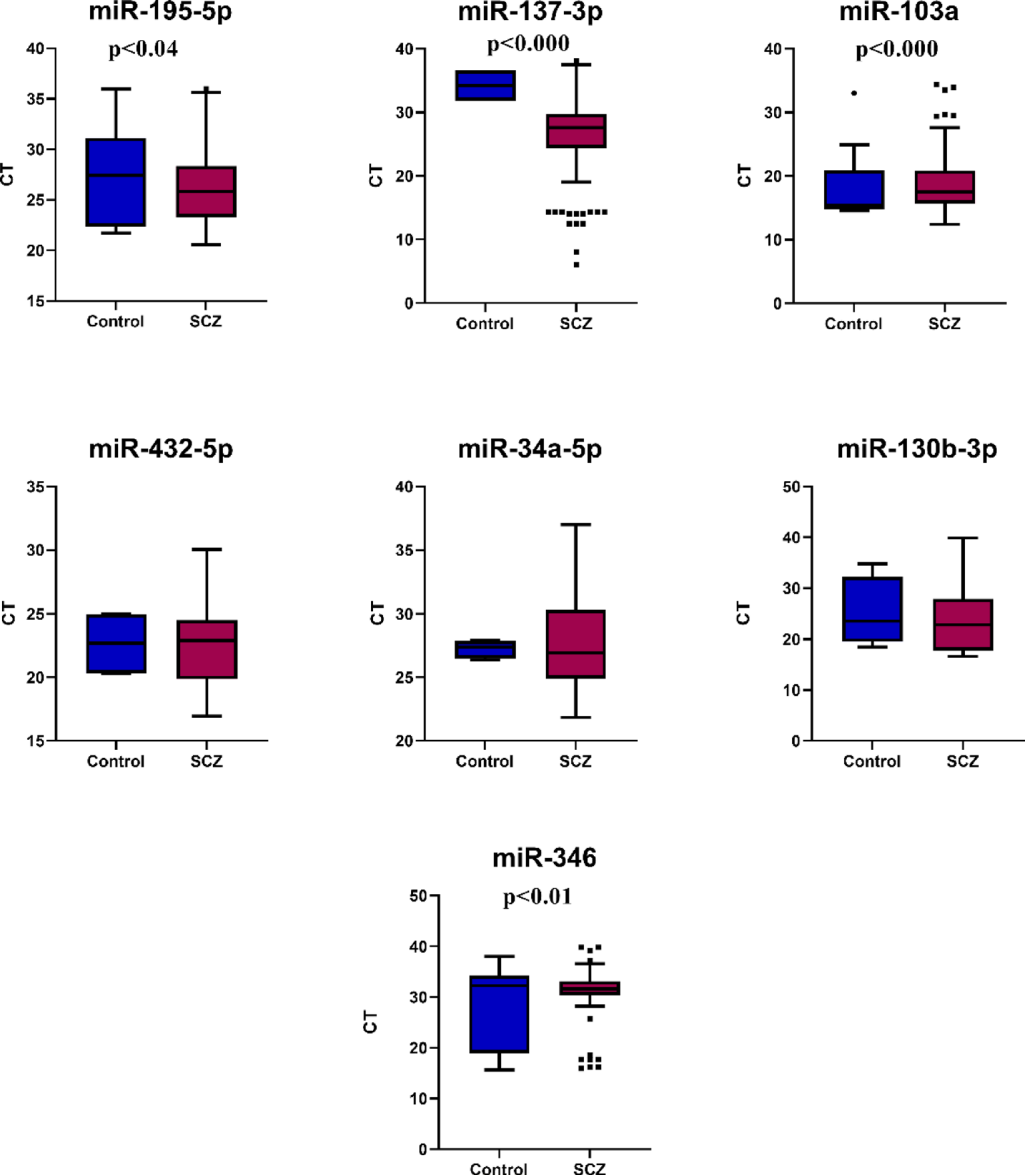
The expression profiles of various miRNAs (miR-195-5p, miR-137-3p, miR-103a, miR-432-5p, miR-34a-5p, miR-130b-3p, and miR-346) in schizophrenia (SCZ) patients compared to controls. Significant differences in expression levels are observed for miR-195-5p, miR-137-3p, miR-346, and miR-103a, as indicated by their respective p-values. Each box plot illustrates the range of cycle threshold (CT) values for miRNAs, with outliers marked for specific cases. These findings highlight miRNA dysregulation in schizophrenia, suggesting their potential role in disease pathology.

In this study, the expression levels of seven miRNAs were assessed using qPCR in schizophrenia patients and a healthy control group. Results indicated changes in mean ± SD of circulating miRNAs among control and schizophrenia cases. There were highly significant differences in the expression levels of miR-137-3p (upregulation compared to control), miR-103a-3p (downregulation compared to control), as well as significant differences in miR-346 (downregulation compared to control) and miR-195-5p (upregulation compared to control) (*p*-value of 0.000, 0.000, 0.01, and 0.04 respectively) (Table 2) and Figs. 2 and 3**).**

**Table 2:** Differences between the studied groups according to their MiRNA levels.

	Cases		Control		Mann whitney	*p*-value	BH adjusted *p* value
	Median	IQR	Median	IQR			
miR-195-5p mean ± SD	25.95	3.14	27.44	1.45	**St.t test=** 2.36	.**022** **(S)**	**0.04** **(S)**
miR-432-5p mean ± SD	22.46	3.06	22.64	2.38	**St.t test=** 0.203	0.840	0.84
miR-346	31.50	29.47–32.95	25.05	17.84–32.26	3.62	**0.004** **(HS)**	**0.01** **(S)**
miR-130b-3p	22.80	17.74–27.90	25.08	23.56–26.61	1.523	0.128	0.18
miR-137-3p	27.49	24.30-29.48	34.22	31.82–36.61	5.262	**0.000** **(HS)**	**0.00** **(HS)**
miR-34a-5p	26.86	25.06–30.46	27.25	26.65–27.85	0.415	0.678	0.79
miR-103a-3p	17.42	15.70-20.93	15.16	14.91–15.42	3.18	**0.000** **(HS)**	**0.00** **(HS)**

S: significant p˂0.05; HS: Highly significant p˂0.01; IQR: Inter Quartile Range; SD: Standard Deviation.

**Fig. 4:**
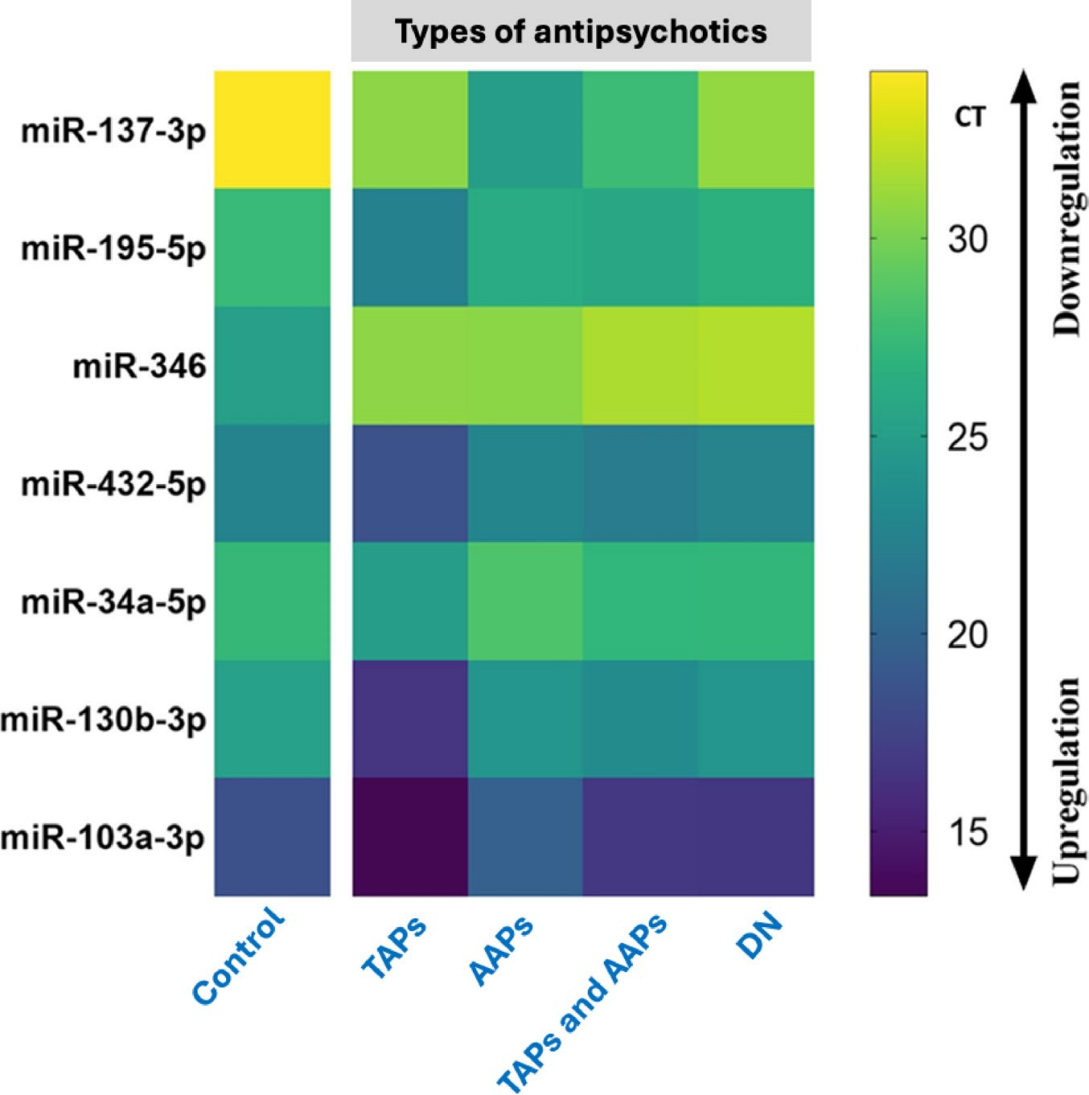
Heatmap illustrating the expression levels of various miRNAs (miR-137-3p, miR-195-5p, miR-346, miR-432-5p, miR-34a-5p, miR-130b-3p, and miR-103a-3p) across different groups based on antipsychotic treatment: Control, TAPs (Typical Antipsychotics), AAPs (Atypical Antipsychotics), TAPs and AAPs combined, and DN (Drug-Naïve). The color scale represents cycle threshold (CT) values, with yellow indicating downregulation (higher CT) and purple indicating upregulation (lower CT). The data reveal distinct miRNA expression patterns depending on the type of antipsychotic treatment, highlighting the potential regulatory effects of antipsychotics on miRNA expression in schizophrenia.

The effect of medication on the expression level of the miRNAs was also studied. The medications were grouped into four clusters based on the type of antipsychotic drugs administered to the schizophrenia cases. Cluster 1 comprised patients for whom Typical Antipsychotics were prescribed, representing 2.5%. Cluster 2 consisted of patients for whom Atypical Antipsychotics were prescribed, representing 70%. Cluster 3 included patients for whom both Typical Antipsychotics and Atypical Antipsychotics were prescribed, representing 17.5%. Finally, cluster 4 comprised drug-naive patients, representing 10% of the cohort. These clusters were compared with the seven miRNAs’ cycle threshold (CT) points. A CT above 30 was colored yellow, indicating downregulation of the miRNA, while a CT below 15 was colored dark blue, indicating upregulation of the miRNA (Fig. 4).

**Fig. 5:**
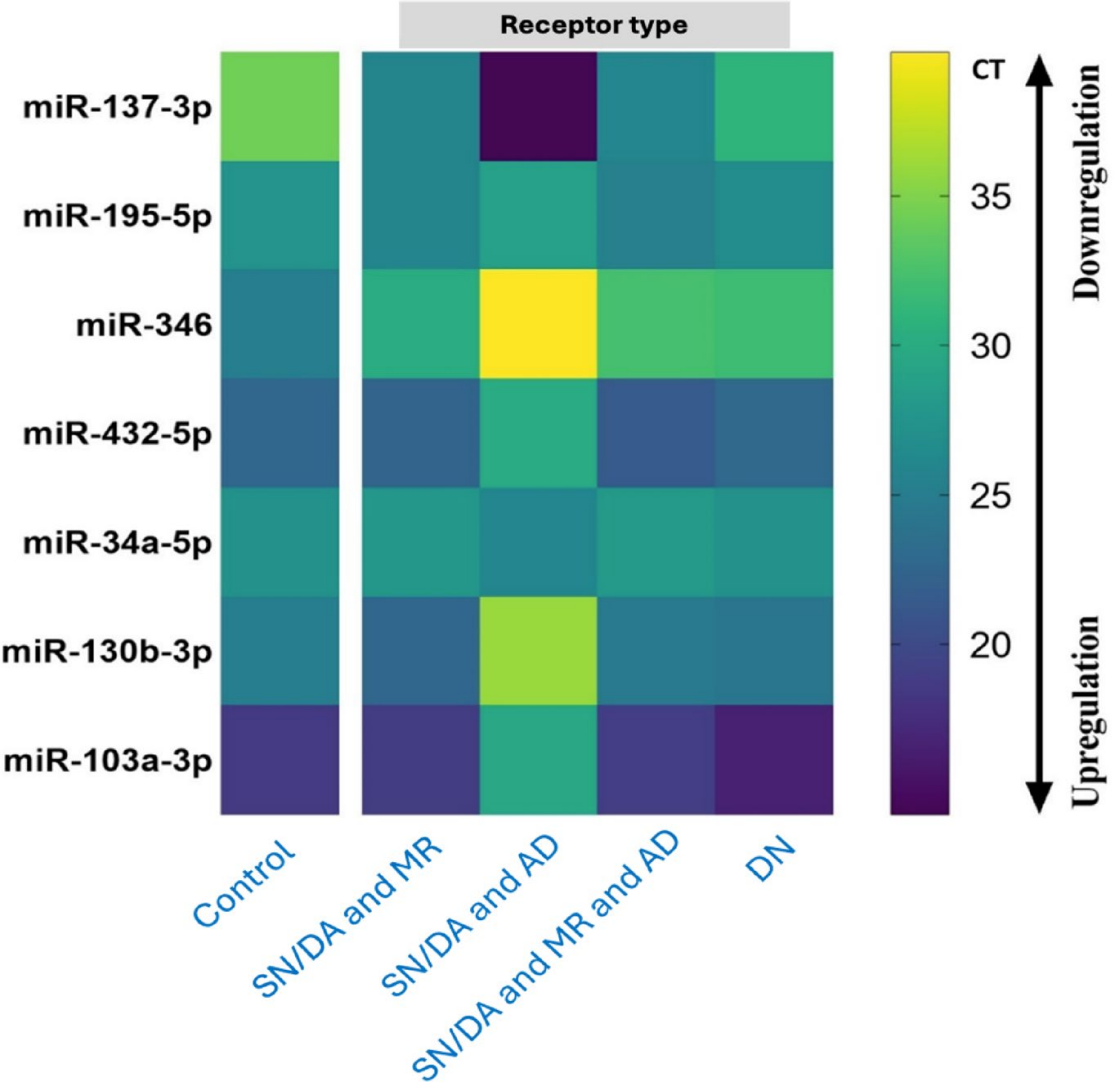
Heatmap showing the expression levels of miRNAs (miR-137-3p, miR-195-5p, miR-346, miR-432-5p, miR-34a-5p, miR-130b-3p, and miR-103a-3p) across different receptor types in schizophrenia patients: Control, SN/DA and MR (Serotonergic/Dopaminergic and Muscarinic Receptors), SN/DA and AD (Adrenergic Receptors), SN/DA and MR and AD, and DN (Drug-Naïve). The color gradient represents CT values, where yellow indicates downregulation (higher CT), and purple indicates upregulation (lower CT). Distinct expression patterns are observed across receptor types, with significant variability in miRNA levels, suggesting receptor-specific regulatory effects and the influence of treatment on miRNA expression profiles.

The medications were categorized into four clusters based on the receptor-based antipsychotic drugs taken by our schizophrenia cases. Cluster 1 included patients for whom Serotonergic/Dopaminergic and Muscarinic receptor antipsychotics were prescribed, representing 70%. Cluster 2 consisted of patients for whom Serotonergic/Dopaminergic and Adrenergic receptor antipsychotics were prescribed, representing 2.5%. Cluster 3 comprised patients for whom Serotonergic/Dopaminergic, Muscarinic, and Adrenergic receptors antipsychotics were prescribed, representing 17.5%. Finally, cluster 4 consisted of drug-naive patients, representing 10% of the cohort. These clusters were compared with the seven miRNAs’ cycle threshold (CT) points. A CT above 35 was colored yellow, indicating downregulation of the miRNA, while a CT below 20 was colored dark blue, indicating upregulation of the miRNA (Fig. 5).

### Questionnaires analysis

#### General health questionnaire-12

**Fig. 6:**
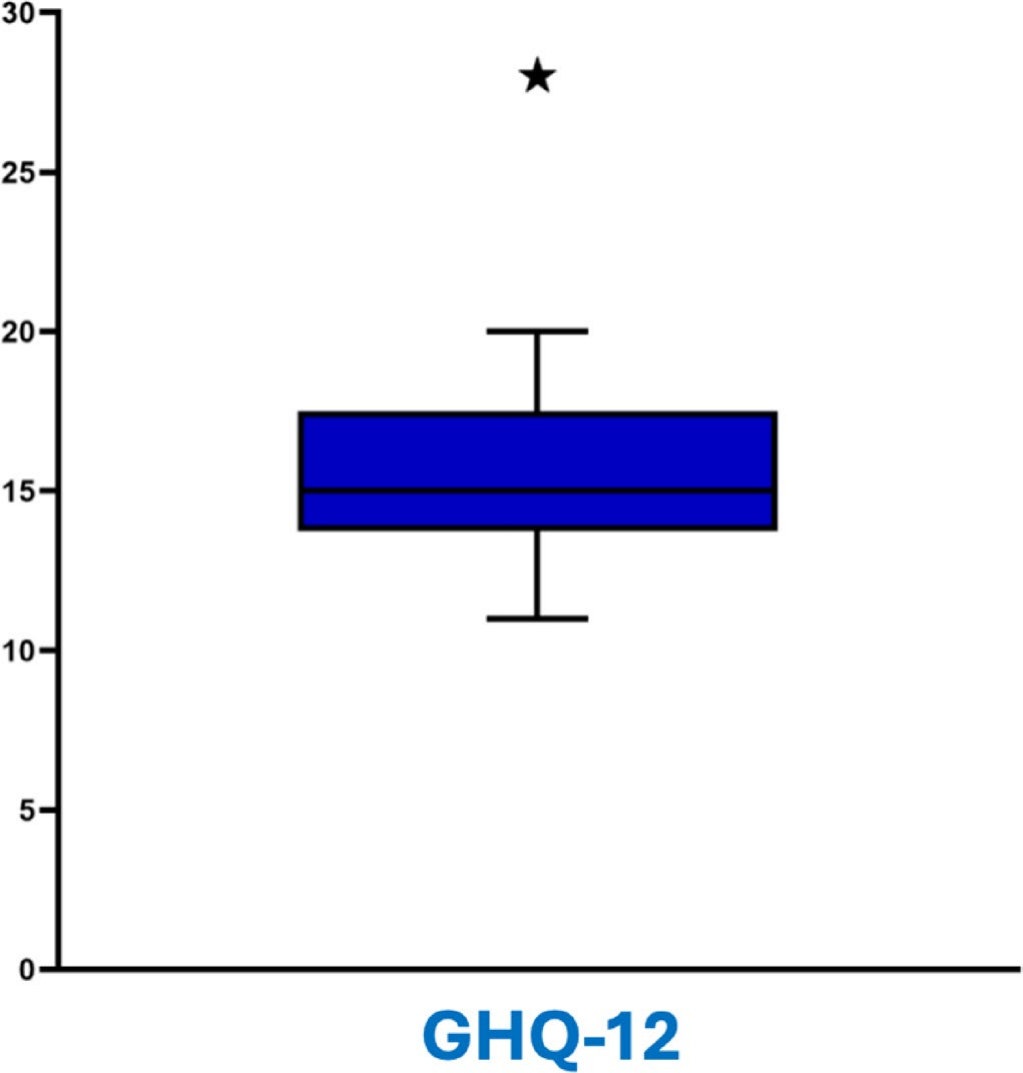
Boxplot representing the General Health Questionnaire-12 (GHQ-12) scores in the studied group. The median score lies within the interquartile range, indicating a moderate level of psychological distress among most individuals. The whiskers represent the range of typical scores, while a single outlier above 25 suggests significantly higher psychological distress in one individual. This visualization highlights the overall distribution and variability of GHQ-12 scores in the population.

Each of the 12 items is rated on a 4-point scale from 0 to 3, using the Likert scoring method, resulting in a total score ranging from 0 to 36. In our control cases, the median GHQ-12 score was 15, with an interquartile range of 13.75–17.5. Scores below 15 are generally considered non-distressed, while scores between 15 and 18 suggest mild distress. Scores above 18 indicate significant distress (Fig. 6).

## Positive and negative syndrome scale

The assessment evaluates three domains of symptoms for our schizophrenia cases: positive, negative, and general psychopathology. The positive scale consists of 7 items (delusions, conceptual disorganization, hallucinations, excitement, grandiosity, suspiciousness/persecution, and hostility) with a median score of 14 and an interquartile range of 10.50–18.50. The negative scale consists of 7 items (blunted affect, emotional withdrawal, poor rapport, passive/apathetic social withdrawal, difficulty in abstract thinking, lack of spontaneity and flow of conversation, and stereotyped thinking) with a median score of 24 and an interquartile range of 20-27.50.

**Fig. 7:**
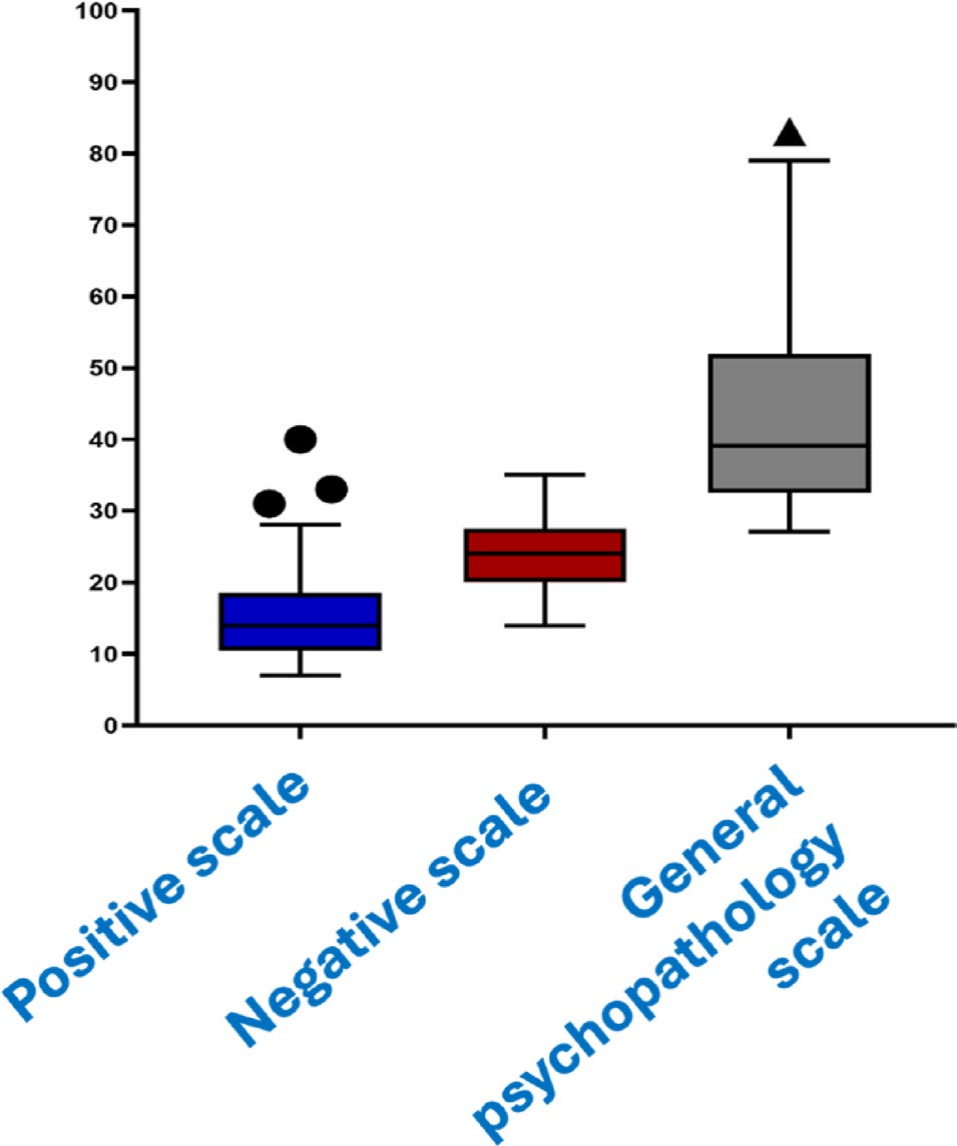
Boxplot comparison of Positive, Negative, and General Psychopathology scales in schizophrenia patients. The Positive scale shows the lowest scores with limited variability, while the Negative scale indicates slightly higher scores with moderate variability. The General Psychopathology scale displays the highest scores with substantial variability, including outliers, suggesting a more widespread impact of general psychopathology symptoms compared to positive and negative symptoms in the studied population. This highlights the heterogeneity in symptom severity among schizophrenia patients.

The general psychopathology scale consists of 16 items (somatic concern, anxiety, guilt feelings, tension, mannerisms and posturing, depression, motor retardation, uncooperativeness, unusual thought content, disorientation, poor attention, lack of judgment and insight, disturbance of volition, poor impulse control, preoccupation, and active social avoidance) with a median score of 39 and an interquartile range of 32.50–52. The PANSS total score ranges from 30 to 210, with higher scores indicating greater symptom severity (Fig. 7).

### Wechsler adult intelligence scale III and trial making test B

**Fig. 8:**
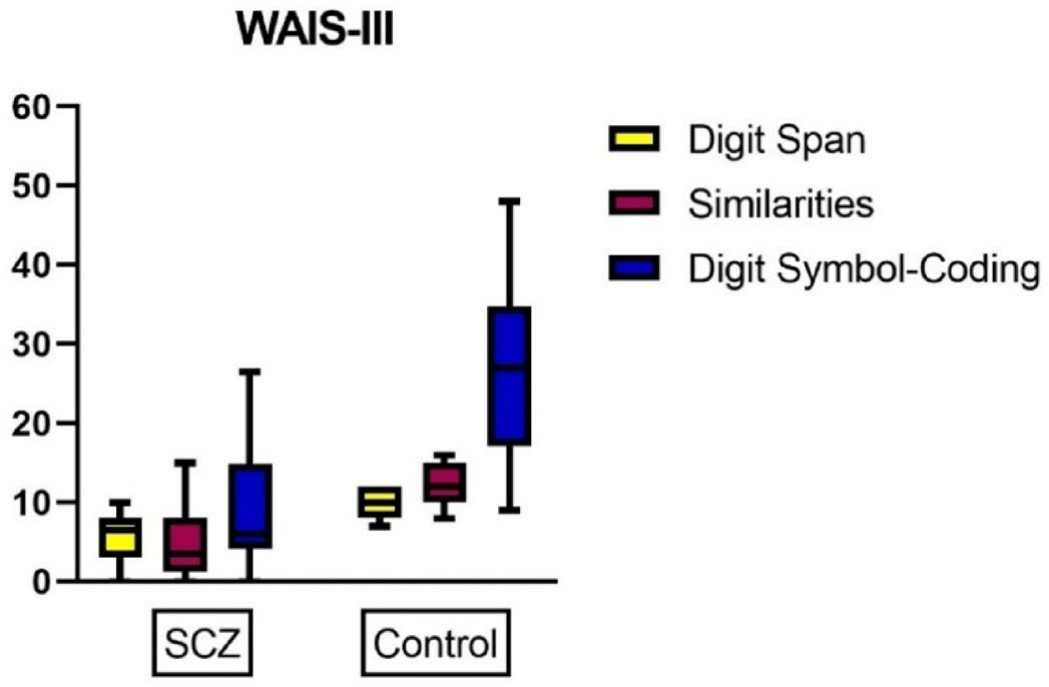
Boxplot showing differences between the schizophrenia cases and control group according to their WAIS-III.

**Fig. 9:**
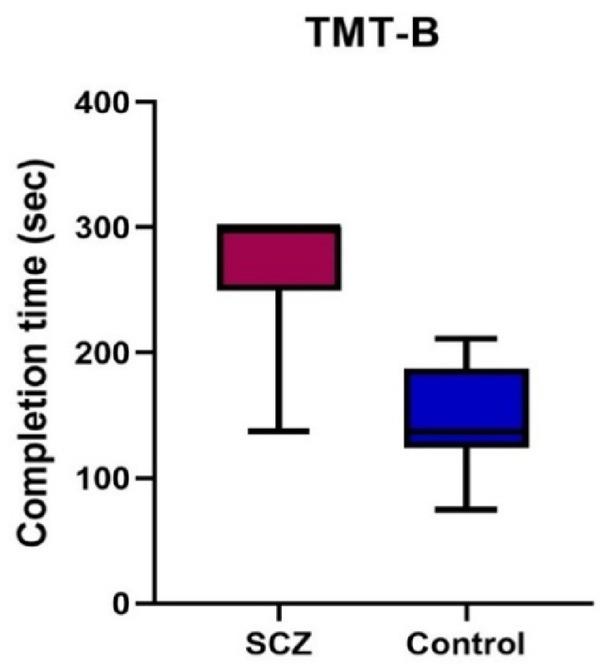
Boxplot showing differences between the schizophrenia cases and control group according to their TMT-B.

Our study utilized the WAIS-III to evaluate a range of cognitive abilities, focusing on three specific subtests. The first subtest, digit span, assesses working memory; the second subtest, similarities, assesses verbal comprehension; and the third subtest, digit symbol coding, evaluates the processing speed of visual information. When comparing the results of the schizophrenia cases and the control group, we found a significant difference in performance. Additionally, we employed the TMT-B to assess executive functioning, cognitive flexibility, and task switching. Similar to the WAIS-III results, we observed a high significance between the schizophrenia cases and the control group. Notably, longer completion times in these tests were indicative of an impaired cognitive function Table (3) and Figs. (8, 9).

**Table 3:** Differences between the studied groups according to their WAIS-III and TMT-B.

	Cases Median IQR		Control Median IQR		Mann Whitney	*p*-value
Digit Span	6.50	3.00–8.00	10.00	8.00–12.00	**4.297**	**0.000 (HS)**
Similarities	3.50	1.25-8.00	12.00	10.00–15.00	**4.437**	**0.000 (HS)**
Digit symbol coding	6.00	4.12–14.87	27.00	17.12–34.75	**4.516**	**0.000 (HS)**
TMT B completion time (Sec)	300.00	249.30–300.00	136.80	124.20-187.05	**5.199**	**0.000 (HS)**

HS: Highly significant p˂0.01; IQR: Inter Quartile Range.

**Wisconsin Card Sorting Test**.

Both groups underwent the Wisconsin Card Sorting Test to evaluate executive function and cognitive flexibility. The following key metrics were used for analysis: Categories Completed (CC) represents the number of completed categories, reflecting cognitive flexibility and the ability to adapt to changing rules. Higher CC scores are more favorable. Categories Experienced (CE) includes completed categories and those abandoned due to errors, providing additional context beyond CC. A higher CE suggests better overall performance. Total Errors (TE) combines all types of errors (perseverative, non-perseverative, and conceptual). Elevated TE may indicate difficulties in rule shifting or attentional lapses. CC, CE, and TE showed a highly significant difference between the control group and schizophrenia cases.

**Fig. 10:**
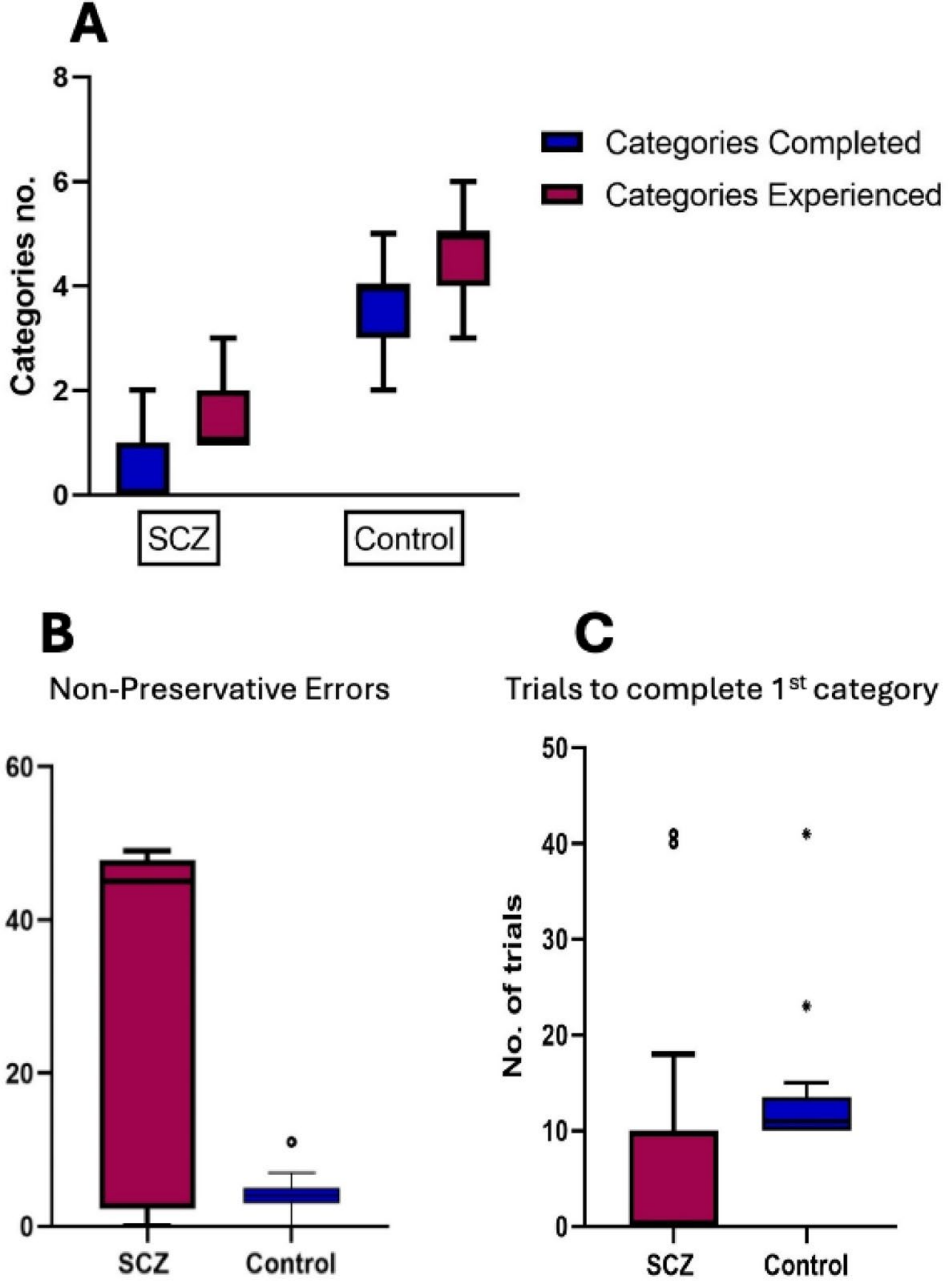
Comparative analysis of cognitive performance between schizophrenia (SCZ) patients and control groups. (**A**): The number of categories completed and experienced shows significantly reduced performance in the SCZ group compared to controls, indicating impaired cognitive flexibility. (**B**): Non-perseverative errors are markedly higher in the SCZ group, reflecting greater difficulty in error monitoring and correction. (**C**): The number of trials required to complete the first category is significantly higher in SCZ patients, demonstrating delayed problem-solving abilities. These findings highlight notable cognitive impairments in schizophrenia patients compared to healthy individuals.

Furthermore, specific error types were considered: Perseverative Responses (PR) are incorrect responses that persistently follow an old sorting rule. Elevated PR may suggest frontal lobe dysfunction. Perseverative Errors (PE) specifically refer to errors related to perseveration, reflecting impaired set-shifting ability. Non-Perseverative Errors (NPE) are errors unrelated to perseveration and may reflect other cognitive difficulties, such as attention deficits or impulsivity. NPEs were significantly different between both groups. Additional metrics include Trials to Complete the First Category (TCFC), where the number of trials needed to complete the initial category. Lower TCFC indicates better cognitive flexibility. Failure to Maintain Set (FMS) where elevated FMS suggests difficulties in maintaining rules. For conceptual Level Responses (CLR), higher scores indicate better abstract reasoning. TCFC, FMS, and CLR were highly significant between the control group and schizophrenia cases (Table 4; Fig. 10).

### Correlation between MiRNAs and different variables

Multivariate binary logistic regression analysis was done to predict schizophrenia cases. The model revealed that one unit increase in miRNA346 was associated with 1.180 increase of probability of schizophrenia cases (OR = 1.180, 95% CI = 1.054–1.321, *P* = 0.004), one unit increase in miRNA137 was associated with 0.380 increase of probability of schizophrenia cases (OR = 0.380, 95% CI = 0.190–0.757, *P* = 0.006), one unit increase in miRNA103a was associated with 1.707 increase of probability of schizophrenia (OR = 1.707, 95% CI = 1.076–2.707, *P* = 0.023), (Table 5).

**Table 4:** Multivariate logistic regression analysis to predict schizophrenia cases.

Variable	B (95% CI) †	*P*-value
miRNA195	0.819 (0.650-1.033)	0.091
miRNA432	0.976 (0.789-1.207)	0.852
miRNA346	1.180 (1.054–1.321)	0.004*
miRNA130b	0.947 (0.846-1.059)	0.341
miRNA137	0.380 (0.190-0.757)	0.006*
miRNA34a	1.058 (0.854 − 1.310)	0.608
miRNA103a	1.707 (1.076–2.707)	0.023*

*Significant P-value; †Adjusted for Sociodemographic; B: Regression coefficient; CI: Confidence interval.

The Spearman correlation analysis revealed several key findings regarding the relationship between miR-195-5p expression and cognitive tests.

No significant correlations were observed between miR-195-5p and various cognitive measures from the Wisconsin Card Sorting Test (WCST), such as categories completed and total errors.

**Table 5:** Differences between the studied groups according to their WCST.

Variable	Cases Median IQR		Control Median IQR		Mann Whitney	*p*-value
Categories completed	0.00	0.00–1.00	4.00	3.00–4.00	5.781	**0.000 (HS)**
Categories experienced	1.00	1.00–2.00	5.00	4.00–5.00	5.786	**0.000 (HS)**
Total errors	45.00	40.00-47.75	12.00	10.00-14.25	5.548	**0.000 (HS)**
Perseverative responses	0.00	0.00–50.00	19.50	17.50–23.00	1.551	0.121
Perseverative errors	0.00	0.00–37.00	8.00	5.00–10.00	1.262	0.207
Non-perseverative errors	45.00	2.25–47.75	4.00	3.00–5.00	2.222	**0.026 (S)**
Unique errors	0.00	0.00–1.00	0.00	0.00–1.00	0.615	0.539
Trails to complete the first category	0.00	0.00–10.00	11.00	10.00-13.50	3.491	**0.000 (HS)**
Failure to maintain a set	0.00	0.00–0.00	1.00	0.00–2.00	4.030	**0.000 (HS)**
Conceptual level responses	6.00	3.00–13.00	49.5	46.5–55.00	5.627	**0.000 (HS)**

HS: Highly significant p˂0.01; IQR: Inter Quartile Range.

Similarly, no significant correlations were found with the Positive and Negative Syndrome Scale (PANSS) subscales, including the positive, negative, and general psychopathology scales as well as WAIS-III subtests.

**Fig. 11:**
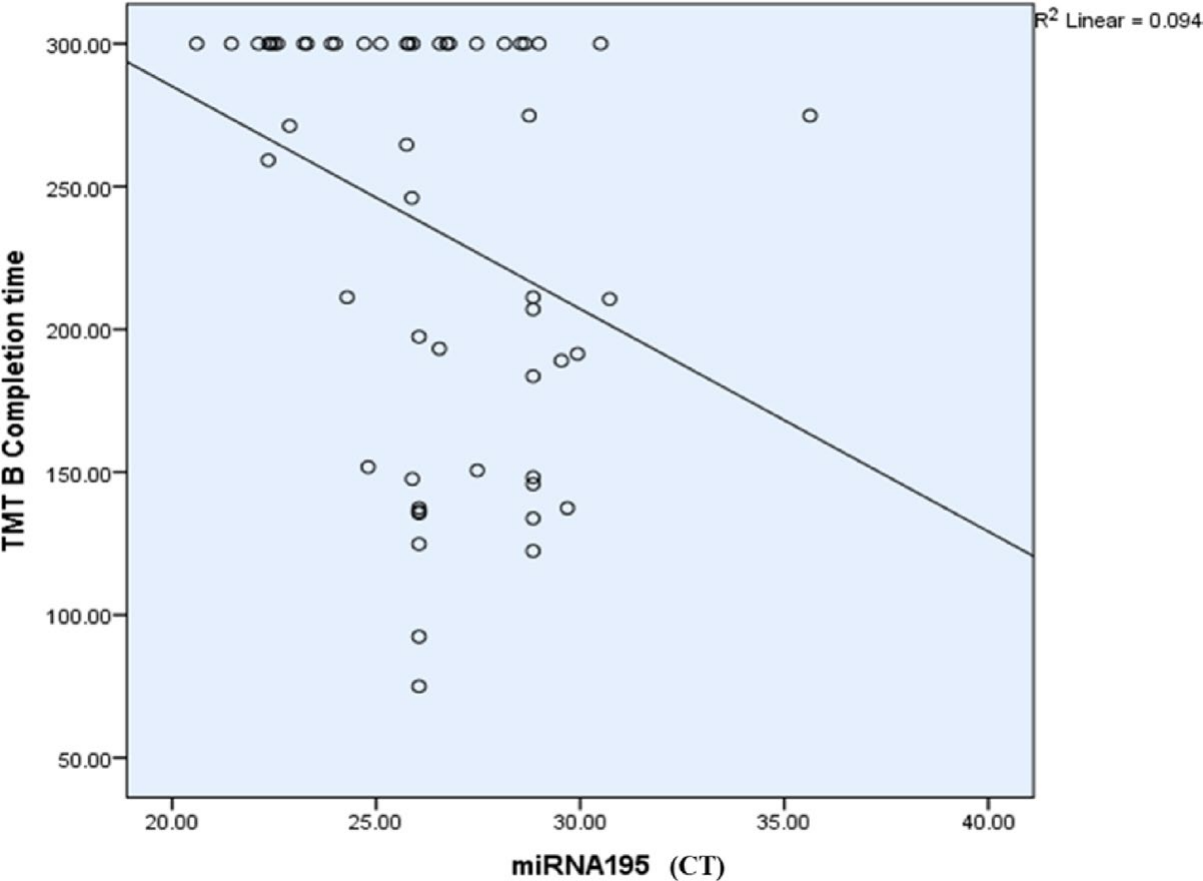
Scatter plot showing the relationship between miR-195 (CT values) and TMT-B (Trail Making Test Part B) completion time in schizophrenia patients. The negative slope of the regression line indicates an inverse relationship, where higher miR-195 expression (lower CT values) is associated with shorter TMT-B completion times, reflecting better cognitive flexibility. However, the R^2^ value (0.094) suggests a modest correlation between miR-195 levels and cognitive performance.

However, a highly significant inverse correlation was observed between miR-195-5p expression and the completion time for the Trail Making Test Part B (TMT-B). This suggests that with the upregulation of miR-195 (indicated by a decrease in CT), the time required to complete the TMT-B increases, which may reflect a slower cognitive processing speed (Table 6; Fig. 11**)**.

**Table 6:** Correlation between miR-195-5p and different variables.

miR-195-5p	Spearman correlation coefficient	*p*-value	BH-adjusted *p*-values
Categories completed	0.080	0.563	0.90
Categories experienced	0.077	0.580	0.77
Total errors	− 0.078	0.573	0.86
Perseverative responses	− 0.218	0.112	0.54
Perseverative error	− 0.218	0.112	0.38
Non-perseverative error	0.216	0.116	0.31
Unique errors	0.268	0.050	0.40
Trails to complete the first category	0.033	0.813	0.85
Failure to maintain a set	0.205	0.137	0.27
Conceptual level responses	0.043	0.756	0.86
TMT B completion time	− 0.392	**0.000 (HS)**	**0.000 (HS)**
Digit Span	0.172	0.214	0.37
Similarities	0.249	0.07	0.42
Digit symbol coding	0.280	**0.040 (S)**	0.48
Positive scale	− 0.277	0.118	0.26
Negative scale	− 0.096	0.594	0.75
General psychopathology scale	− 0.225	0.208	0.38

S: Significant p˂0.05; HS: Highly significant p˂0.01.

Based on the analysis using the Spearman correlation coefficient, no statistically significant correlations were observed between miR-432-5p and variables such as PANSS (Positive and Negative Syndrome Scale), WCST (Wisconsin Card Sorting Test), and WAIS-III (Wechsler Adult Intelligence Scale-III). However, a negative trend was noted between miR-432-5p and perseverative responses in the WCST (*r* = -0.230, *p* = 0.095) and the general psychopathology scale of PANSS (*r* = -0.228, *p* = 0.202). Additionally, a positive trend was identified between miR-432-5p and non-perseverative errors in the WCST (*r* = 0.212, *p* = 0.124). These relationships, though not statistically significant, suggest potential associations that may warrant further investigation (Table 7).

**Table 7:** Correlation between miR-432-5p and different variables.

miR-432-5p	Spearman correlation Coefficient	*p*-value
Categories completed	− 0.024	0.863
Categories experienced	− 0.030	0.831
Total errors	0.025	0.857
Perseverative responses	− 0.230	0.095
Perseverative errors	− 0.191	0.166
Non-perseverative errors	0.212	0.124
Unique errors	0.211	0.126
Trails to complete the first category	0.038	0.786
Failure to maintain a set	0.162	0.242
Conceptual level responses	− 0.109	0.432
TMT B completion time	− 0.063	0.653
Digit Span	− 0.005	0.970
Similarities	0.124	0.373
Digit symbol coding	− 0.022	0.873
Positive scale	0.101	0.320
Negative scale	− 0.178	0.320
General psychopathology scale	− 0.228	0.202

**Fig. 12:**
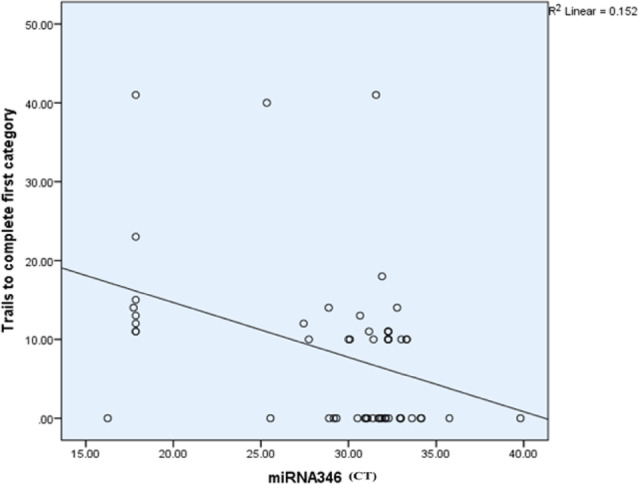
SScatter plots showing the relationship between miR-346 expression (CT values) and cognitive performance measure. The plot depicts a negative relationship between miR-346 CT values and the number of trials required to complete the first category, where higher miR-346 expression is linked to fewer trials (R^2^ = 0.152). These findings suggest that miR-346 may play a role in cognitive functioning as processing speed in schizophrenia patients.

The Spearman correlation coefficient analysis indicated no significant correlations between miR-346 expression and variables such as PANSS (Positive and Negative Syndrome Scale), TMT-B, WAIS-II (Wechsler Adult Intelligence Scale-II), and Wisconsin Card Sorting Test (WCST). Additionally, a significant inverse correlation was observed between miR-346 expression and the number of trials required to complete the first category (*r* = − 0.398, *p* = 0.01, BH-adjusted). The negative slope of the regression line indicates that higher CT values of miR-346 correspond to fewer trials needed to complete the first category. The R² value of 0.152 reflects a modest negative linear relationship. As the miR-346 level is downregulated, the number of trials required to complete the first category decreases (Table 8; Fig. 12).

**Table 8:** Correlation between miR-346 and different variables.

miR-346	Spearman correlation coefficient	*p*-value	BH-adjusted *p*-values
Age	0.053	0.705	0.50
Onset	− 0.018	0.913	10.96
Categories completed	− 0.277	**0.043 (S)**	0.26
Categories experienced	− 0.277	**0.043 (S)**	0.34
Total errors	0.307	**0.024 (S)**	0.12
Perseverative responses	− 0.141	0.309	1.06
Perseverative errors	− 0.182	0.187	0.45
Non-perseverative error	0.182	0.187	0.37
Unique errors	− 0.057	0.682	1.26
Trails to complete the first category	− 0.398	**0.003 (HS)**	**0.01(S)**
Failure to maintain a set	− 0.272	**0.047 (S)**	0.08
Conceptual level responses	− 0.233	0.090	0.14
TMT B completion time	− 0.080	0.567	0.76
Digit Span	− 0.146	0.292	0.37
Similarities	− 0.002	0.987	1.18
Digit symbol coding	− 0.034	0.806	0.19
Positive scale	− 0.080	0.656	0.76
Negative scale	− 0.239	0.180	0.19
General psychopathology scale	− 0.017	0.926	0.93

S: Significant p˂0.05; HS: Highly significant p˂0.01

**Fig. 13:**
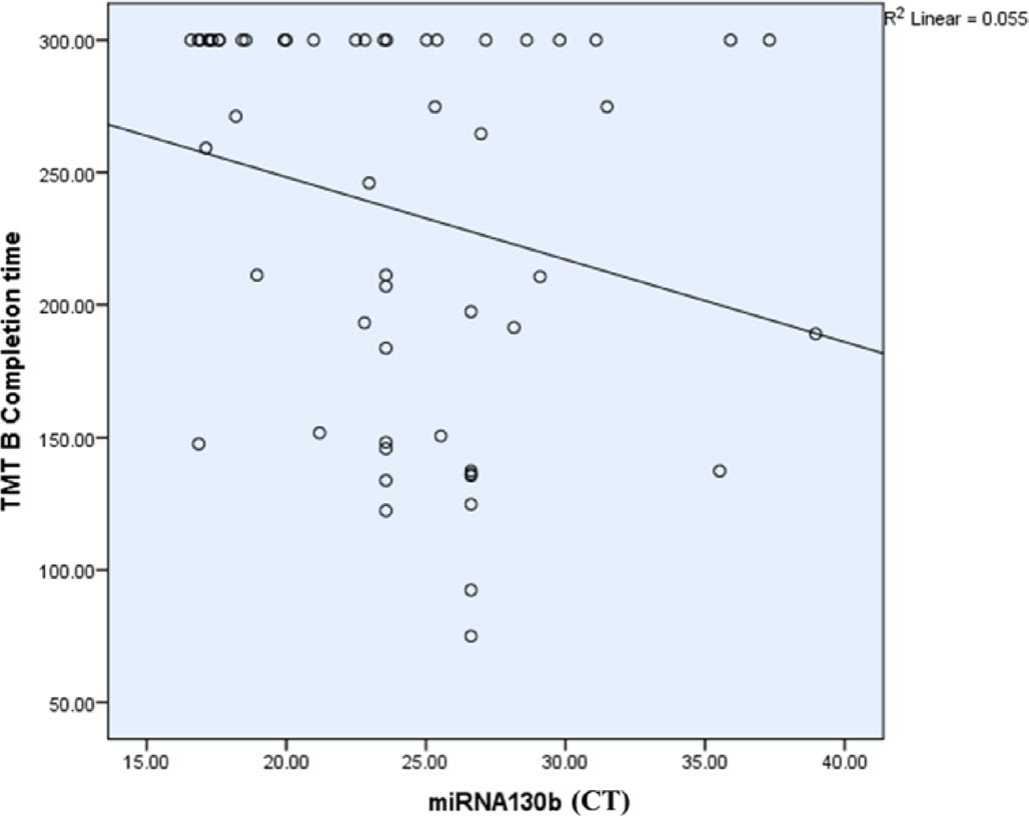
Scatter plot showing the relationship between miR-130b (CT values) and TMT-B (Trail Making Test Part B) completion time in schizophrenia patients. The negative slope of the regression line suggests an inverse correlation, where higher miR-130b expression (lower CT values) is associated with shorter TMT-B completion times, indicating better cognitive flexibility. However, the low R² value (0.055) suggests a weak overall correlation.

The Spearman correlation coefficient analysis did not reveal any significant correlation after BH- adjusted p- values between the expression of miR-130b-3p and variables such as WAIS-III (Wechsler Adult Intelligence Scale-III), WCST (Wisconsin Card Sorting Test, TMT-B (Trail Making Test B) and PANSS, (Table 9; Fig. 13**)**.

**Table 9:** Correlation between miR-130b-3p and different variables.

miR-130b-3p	Spearman correlation coefficient	*p*-value	BH-adjusted *p*-values
Categories completed	0.073	0.599	0.85
Categories experienced	0.073	0.587	0.88
Total errors	− 0.055	0.691	0.87
Perseverative responses	− 0.159	0.252	0.60
Perseverative errors	− 0.180	0.192	0.92
Non-perseverative errors	0.161	0.246	0.84
Unique errors	0.212	0.125	0.75
Trails to complete the first category	− 0.029	0.836	0.84
Failure to maintain a set	0.051	0.712	0.85
Conceptual level responses	0.040	0.772	0.88
TMT B completion time	− 0.298	**0.029 (S)**	0.35
Digit Span	0.078	0.574	0.92
Similarities	0.101	0.468	0.80
Digit symbol coding	0.102	0.461	0.92
Positive scale	− 0.331	0.061	0.49
Negative scale	− 0.180	0.317	0.69
General psychopathology scale	− 0.387	**0.02 (S)**	0.48

**Fig. 14:**
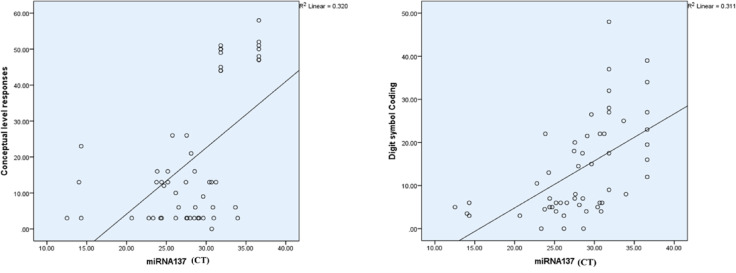
Scatter plots showing the relationship between miR-137 expression (CT values) and cognitive performance measures in schizophrenia patients. Left Plot: A positive correlation (R^2^ = 0.320) is observed between miR-137 CT values and conceptual level responses, suggesting that higher miR-137 expression (lower CT values) is associated with improved abstract thinking and problem-solving abilities. Right Plot: A similar positive correlation (R^2^ = 0.311) is evident between miR-137 CT values and digit symbol coding performance, indicating that increased miR-137 expression corresponds to better processing speed and cognitive function. These findings highlight the potential role of miR-137 in regulating cognitive performance in schizophrenia.

The Spearman correlation coefficient analysis revealed no significant associations between miR-137-3p expression and clinical variables such as PANSS scores. However, a highly significant positive correlation was observed between miR-137-3p expression and specific metrics from the Wisconsin Card Sorting Test (WCST), including categories completed, categories experienced, total errors, conceptual level responses, and failure to maintain a set. Furthermore, miR-137-3p expression demonstrated strong correlations with cognitive performance on the Trail Making Test Part B (TMT-B) and two subtests of the WAIS-III, namely digit symbol coding and similarities. Notably, both conceptual level responses and digit symbol coding scores exhibited a directly proportional relationship with miR-137-3p expression, indicating that the upregulation of miR-137-3p (reflected by a decrease in CT) was associated with a reduction in the performance on these cognitive tasks (Table 10; Fig. 14**)**.

**Table 10:** Correlation between miR-137-3p and different variables.

miR-137-3p	Spearman correlation coefficient	*p*-value	BH-adjusted *p*-values
Categories completed	0.501	**0.000 (HS)**	**0.000 (HS)**
Categories experienced	0.503	**0.000 (HS)**	**0.000 (HS)**
Total errors	− 0.525	**0.000 (HS)**	**0.000 (HS)**
Perseverative responses	0.071	0.610	0.81
Perseverative errors	0.031	0.824	0.94
Non- perseverative errors	− 0.189	0.171	0.29
Unique errors	− 0.175 0.215	0.206	0.33
Trails to complete the first category		0.119	0.24
Failure to maintain a set	0.348	**0.010 (S)**	**0.000 (HS)**
Conceptual level responses	0.520	**0.000 (HS)**	**0.000 (HS)**
TMT B completion time	− 0.733	**0.000 (HS)**	**0.000 (HS)**
Digit Span	0.259	0.059	0.13
Similarities	0.375	**0.000 (HS)**	**0.000 (HS)**
Digit symbol coding	0.651	**0.000 (HS)**	**0.000 (HS)**
Positive scale	− 0.068	0.706	0.89
Negative scale	0.036	0.841	0.92
General psychology scale	0.032	0.861	0.90

**Fig. 15:**
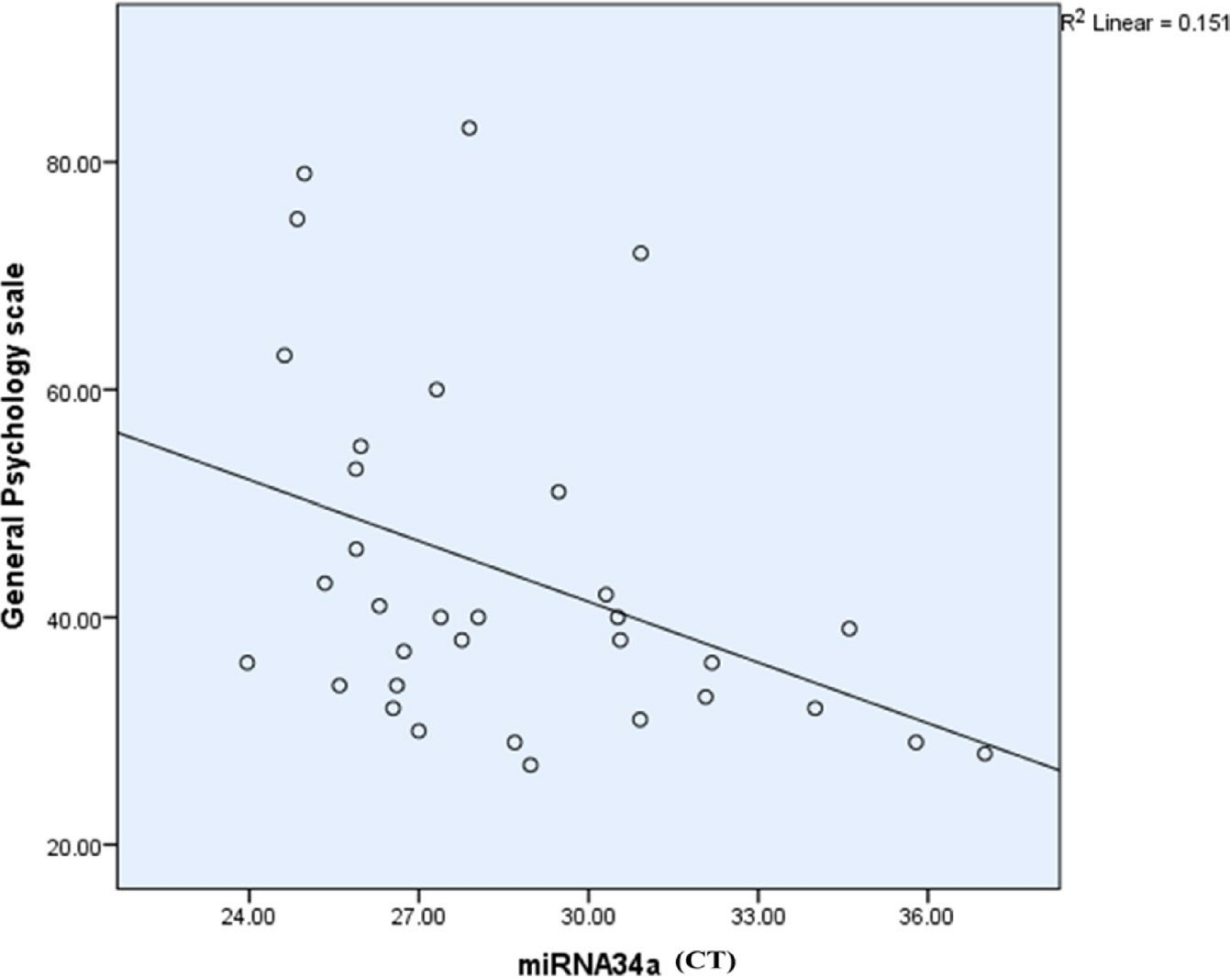
Scatter plot illustrating the relationship between miR-34a expression (CT values) and the General Psychopathology Scale in schizophrenia patients. A negative correlation (R^2^ = 0.151) is observed, indicating that higher miR-34a expression (lower CT values) is associated with lower scores on the General Psychopathology Scale, reflecting reduced severity of general psychopathology symptoms. While the trend is evident, the modest R^2^ value suggests that additional factors may contribute to variability in general psychopathology symptoms.

The Spearman correlation analysis indicated no significant associations between miR-34a-5p expression and variables such as TMT-B performance, WAIS-III, or WCST measures. However, a highly significant negative correlation was found with the general psychopathology scale of PANSS (*r* = – 0.447, *p* = 0.00). These results suggest that upregulation (reflected by a decrease in CT) of miR-34a-5p is associated with higher scores in general psychopathology scales, indicating worsened mental health outcomes in schizophrenia patients (Table 11; Fig. 15).

**Table 11:** Correlation between miR-34a-5p and different variables.

miR-34a-5p	Spearman correlation coefficient	*p*-value	BH-adjusted *p*-values
Categories completed	− 0.006	0.966	7.73
Categories experienced	− 0.008	0.962	5.77
Total errors	0.047	0.735	2.92
Perseverative responses	− 0.146	0.292	1.00
Perseverative error	− 0.127	0.358	0.86
Non-perseverative error	0.135	0.331	0.72
Unique errors	0.155	0.262	0.48
Trails to complete the first category	− 0.030	0.832	1.43
Failure to maintain a set	0.086	0.535	0.86
Conceptual level responses	− 0.094	0.500	0.71
TMT B completion time	− 0.207	0.133	0.18
Digit Span	− 0.026	0.853	1.08
Similarities	0.136	0.326	0.39
Digit symbol coding	0.003	0.983	1.12
Positive scale	− 0.356	**0.042 (S)**	0.50
Negative scale	− 0.319	0.071	0.57
General psychology scale	− 0.447	**0.005 (HS)**	**0.00(HS)**

**Fig. 16:**
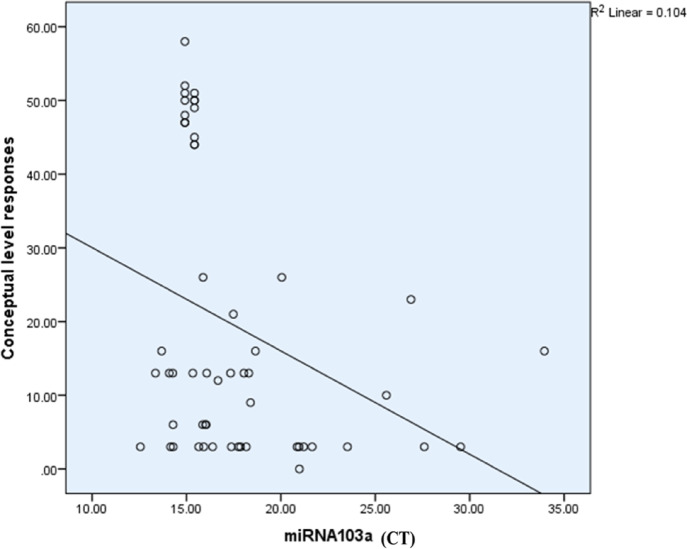
Scatter plot showing the relationship between miR-103a expression (CT values) and conceptual level responses in schizophrenia patients. A negative correlation (R^2^ = 0.104) is observed, where higher miR-103a expression (lower CT values) is associated with improved conceptual level responses, indicative of better abstract thinking and cognitive flexibility. However, the low R² value suggests that miR-103a contributes modestly to variability in conceptual performance, with other factors likely playing a significant role.

**Fig. 17:**
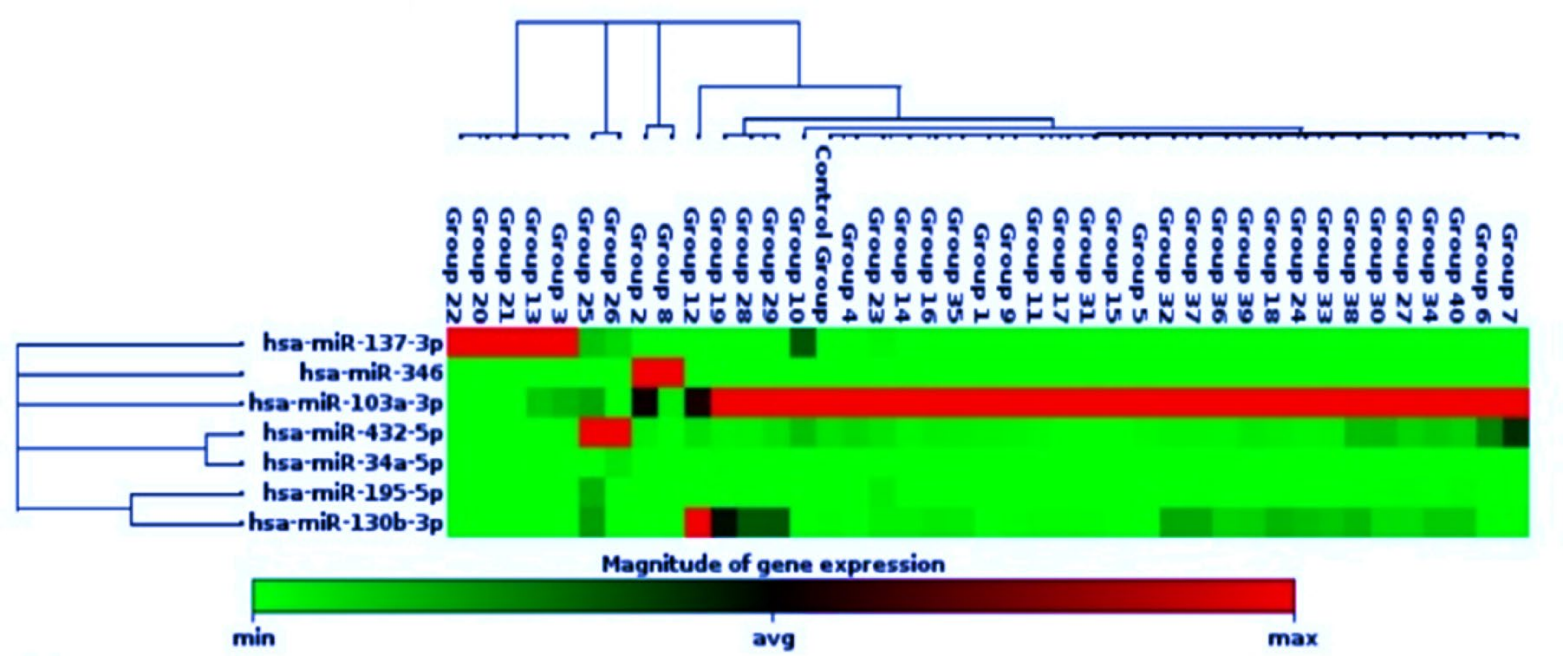
Heatmap illustrating the expression levels of miRNAs (hsa-miR-137-3p, hsa-miR-346, hsa-miR-103a-3p, hsa-miR-432-5p, hsa-miR-34a-5p, hsa-miR-195-5p, and hsa-miR-130b-3p) in schizophrenia patients across different groups. The color scale represents the magnitude of gene expression, ranging from low (green) to high (red), with average levels in black. Distinct clustering patterns are evident, highlighting variations in miRNA expression between patients and controls. These findings emphasize the role of dysregulated miRNAs in schizophrenia pathology.

The Spearman correlation coefficient analysis revealed no significant association between miR-103a-3p expression and some clinical variables such as PANSS, WAIS-II, and TMT-B. However, significant correlations were identified for categories completed, categories experienced, total errors, and conceptual level responses. Notably, an inverse relationship was found between conceptual level responses and miR-103a-3p expression, indicating that downregulation of the miR-103a-3p (reflected by an increase in CT), the number of conceptual level responses decreased (Table 12; Fig. 16). The overall expression profile of different miRNAs is presented in Fig. (17).

**Table 12:** Correlation between miR-103a-3p and different variables.

miR-103a-3p	Spearman correlation coefficient	*p*-value	BH-adjusted *p*-values
Categories completed	− 0.376	0.005 **(HS)**	**0.00 (HS)**
Categories experienced	− 0.378	0.005 **(HS)**	**0.02 (S)**
Total errors	0.377	0.005 **(HS)**	**0.03 (S)**
Perseverative responses	− 0.241	0.080	0.16
Perseverative error	− 0.201	0.146	0.22
Non-perseverative error	0.315	**0.020 (HS)**	0.07
Unique errors	0.229	0.096	0.16
Trails to complete the first category	− 0.205	0.137	0.22
Failure to maintain a set	− 0.179	0.196	0.24
Conceptual level responses	− 0.409	0.002 **(HS)**	**0.02 (S)**
TMT B completion time	0.164	0.236	0.27
Digit Span	− 0.273	0.045 **(S)**	0.12
Similarities	− 0.181	0.190	0.24
Digit symbol coding	− 0.257	0.060	0.13
Positive scale	− 0.255	0.153	0.20
Negative scale	− 0.136	0.451	0.45
General psychology scale	− 0.338	0.055	0.13

## Discussion

Schizophrenia (SZ) is one of the most severe, complex mental disorders with unknown etiology. Like other complex disorders, a combination of genetic and environmental factors plays a role in its development, including altering gene expression via epigenetic mechanisms^59,60^.

Most of the identified miRNAs are expressed in the mammalian brain, playing a crucial role in neurodevelopmental signaling, synaptic plasticity, and adult neuronal activity^61^. Disruptions in either the miRNA biogenesis pathway or the function of a single miRNA can potentially induce neurological deficits in both human subjects and animal models^62^.

In the context of SZ, compelling evidence points to the central role of miRNA dysregulation in the pathogenesis of SZ. Initial findings from postmortem brain studies indicate the dysregulation of multiple miRNAs in individuals diagnosed with SZ^27,63^. Furthermore, the sensitivity of brain miRNA levels to environmental factors linked to an elevated risk of SZ adds another layer to the evidence highlighting the central role of miRNA dysregulation in the pathogenesis of this disorder^64^.

miRNAs are found as freely circulated miRNAs in various body fluids, including serum, plasma, cerebrospinal fluid, urine, and saliva, or encapsulated inside exosomes that are selectively released by cells and play a crucial role in intercellular communication^65^. Freely circulated miRNAs and exosome miRNAs have emerged as promising biomarkers for several diseases, such as cancer, atherosclerosis, cardiovascular diseases, autoimmune disorders, metabolic disorders, and neurodegenerative diseases, including SZ, depression, Alzheimer’s disease, bipolar disorder, and Parkinson’s disease^66^. Both exosome and plasma miRNAs have been well-reported as diagnostic and therapeutic biomarkers for SZ in recent years; however, exosome miRNAs tend to be more favorable because they are more stable and present at a higher level within the exosome than other body fluids^17,67^.

In Egypt, the rising number of individuals with SZ underscores the urgency for improved diagnostic strategies. Presently, the diagnosis primarily relies on symptoms and physical signs. Genetic, protein, and biochemical biomarkers are not incorporated into the diagnostic process for SZ due to the limited access to neuronal tissue and many other factors. Thus, utilizing the easily accessible blood/plasma is of crucial interest when dealing with neurodevelopmental/degenerative diseases. To address this limitation, PBMCs levels of miRNAs stand out as promising candidates for such biomarkers due to their involvement in neurodevelopmental processes and their potential to reflect the underlying pathophysiological changes.

Integrating miRNA expression analysis extracted from peripheral blood (plasma or serum) into diagnostic protocols could provide a more thorough and objective approach to identifying SZ, offering the prospect of improved early detection and targeted interventions. Therefore, in this study, we aimed to analyze the expression of selected miRNAs: miR-137-3p, miR-34a-5p, miR-432-5p, miR-130b-3p, miR-346, miR-195-5p, miR-103a-3p, and their possible diagnostic value for SZ. These specific miRNAs were chosen because of their distinct roles in neuronal development and association with neurological diseases.

Regarding the gender effect on schizophrenia disorder characteristics concerning symptomatology, patients with persisting symptoms tended to be male^68^. In the large World Health Organization SOHO study on 17,000 patients in 37 countries, among them North Africa & Middle East, where the response rates varied across regions and were highest in North Africa & Middle East (84.6%)^69^. Men also showed higher levels of negative symptoms^68,70^.

The results of the present study revealed that 40 male patients with SZ disease were middle-aged; the mean age was (mean ± SD: 32.55 ± 8.80) years. This was in agreement with most previous studies, which revealed that SZ is more common in males with a mean age of 29.2 ± 9.8 for the test cohort and 25.0 ± 7.5 for the validation cohort^71^.

Research consistently demonstrates a substantially higher prevalence of smoking among individuals with schizophrenia compared to the general population^72,73^. In a study by Kelly and McCreadie^74^, smoking in schizophrenia was found to be associated with factors such as younger age, male gender, more frequent hospitalizations, and poorer social adjustment in childhood. Their findings indicated that 58% of individuals with schizophrenia were smokers, compared to only 28% of the control group. More recent research by Dickerson, Schroeder^75^ corroborates these findings, reporting smoking rates of 62% in patients with schizophrenia versus 17% in control groups. Notably, 90% of schizophrenia patients who smoke began smoking before the onset of their illness, suggesting that smoking may serve as a risk factor or marker for the disorder^72,76^.

Our study demonstrated a highly significant difference in smoking prevalence between patients with schizophrenia and the control group. Specifically, 85% of the patients with schizophrenia were smokers, compared to 42.9% in the control group. In another approach, the “self-medication hypothesis” posits that nicotine may have a beneficial effect on cognitive impairment and negative symptoms of schizophrenia, potentially through its regulation of dopamine and nicotinic receptor systems^77–79^.

The multivariate binary logistic regression analysis was conducted to evaluate the association between different miRNAs and schizophrenia cases, adjusting for sociodemographic data. This method estimates the Odds Ratio (OR), which quantifies the likelihood of schizophrenia associated with a unit increase in each miRNA level, while the 95% Confidence Interval (CI) and p-values indicate the statistical significance and precision of these estimates. The analysis identified significant associations between specific miRNAs and schizophrenia risk. miRNA-346 (OR = 1.180, *P* = 0.004) and miRNA-103a (OR = 1.707, *P* = 0.023) were associated with an increased probability of schizophrenia, suggesting their potential role as risk factors. In contrast, miRNA-137 (OR = 0.380, *P* = 0.006) showed a protective effect, reducing schizophrenia likelihood.

MicroRNA-195 (miR-195) has emerged as a potential biomarker and therapeutic target in schizophrenia. miR-195 has been identified as a core regulator in an miRNA-transcription factor regulatory network for schizophrenia, interacting with genes involved in signaling pathways and nervous system regulation^41^. Mechanistically, miR-195 regulates target proteins involved in the cell cycle, apoptosis, and proliferation, with WEE1, CDK6, and Bcl-2 confirmed as target genes^80^. These findings highlight the potential of miR-195 as a biomarker and therapeutic target in schizophrenia, particularly for cognitive symptoms and treatment response.

In the present study, we investigated the role of miR-195 in schizophrenia and observed that miR-195 was significantly upregulated in schizophrenia patients (mean ± SD: 25.95 ± 3.14) compared to the control group (mean ± SD: 27.44 ± 1.45) (*p* = 0.04 S, BH-adjusted). Cognitive function was assessed using the Positive and Negative Syndrome Scale (PANSS), the Wisconsin Card Sorting Test (WCST), three subtests of the Wechsler Adult Intelligence Scale-III (WAIS-III), and the Trail Making Test Part B (TMT-B). No significant correlations were found between miR-195 with the Positive and Negative Syndrome Scale (PANSS) subscales, consistent with previous findings Pan, Feng^42^ where no significant correlations were found when measuring clinical symptoms by PANSS total and sub-scores.

We identified a highly significant inverse correlation between miR-195-5p expression and the completion time for the Trail Making Test Part B (TMT-B) (*r* = -0.392, *p* = 0.00, BH-adjusted), indicating that lower miR-195-5p levels are associated with slower cognitive processing speed, suggesting a potential link between miR-195-5p and certain aspects of cognitive performance.

In agreement with Pan, Feng^42^ investigated miR-195. With BDNF mRNA and BDNF protein levels in peripheral blood, miR-195 expression was significantly elevated. Correlates with lower BDNF protein levels and poorer cognitive performance in schizophrenia patients, suggesting a role in cognitive impairment in schizophrenia. Thus, the development of cognition-enhancing treatment for schizophrenia may be considered a miRNA-based strategy.

On the contrary, Huang, Bao^81^ investigated miR-195 levels in peripheral blood mononuclear cells and analyzed them using quantitative real-time polymerase chain reaction (qRT-PCR). No significant differences in miR-195 levels were found between patients and healthy controls (p *>* 0.05). However, they suggested that the miR-195 level may predict symptomatic improvement and olanzapine response in schizophrenia patients, highlighting miR-195 as a potential therapeutic target for antipsychotic interventions. Furthermore, logistic regression analysis demonstrated that baseline miR-195 levels could be a biomarker for olanzapine response. Conversely, Shi, Du^47^ reported that miR-195 expression was significantly downregulated in schizophrenia patients compared to healthy controls (*p* < 0.0001).

In our study, we identified significant upregulation of miR-137-3p in patients with schizophrenia (median: 27.49) compared to the control group (median: 31.82), with a highly significant p-value (*p* = 0.000). Furthermore, we found a strong positive correlation between miR-137-3p expression and performance metrics on the Wisconsin Card Sorting Test (WCST). miR-137-3p expression was also strongly associated with cognitive performance on the Trail Making Test Part B (TMT-B) and two subtests of the WAIS-III, specifically digit symbol coding and similarities. These findings suggest that miR-137 plays a critical role in the etiology of schizophrenia. This brain-enriched microRNA is involved in the regulation of neuronal development, proliferation, and synaptic maturation^82^. Additionally, genome-wide association studies have identified miR-137 as one of the primary risk genes associated with schizophrenia.

Ou, Liu^83^ conducted a meta-analysis that demonstrated a significant association between the rs1625579 miR-137 genetic variant and an increased risk of schizophrenia. Wu, Zhang^84^ provided further evidence indicating that the aberrant expression of miR-137 in the peripheral blood of schizophrenia patients may suggest its potential as a diagnostic biomarker. Collectively, these findings highlight the pivotal role of miR-137 in the pathogenesis of schizophrenia and its potential application in the development of novel diagnostic and therapeutic strategies.

Consistent with the findings of^84–86^, a significant upregulation of miR-137 expression was observed when comparing schizophrenia patients to healthy controls. He, Lozano^87^ also reported an increase in miR-137 expression in the presence of schizophrenia-associated risk alleles in cultured mouse hippocampal neurons. This elevated expression has been linked to alterations in synaptic transmission, synaptogenesis, and synaptic ultrastructure in hippocampal neurons. Furthermore, there is evidence suggesting an increase in MIR137HG expression in the hippocampus of individuals carrying the disease-associated genotype.

On the contrary, Kandratsenka, Nestsiarovich^88^, who examined the associations of rs1625579 with schizophrenia, symptom severity, and cognitive performance using the Positive and Negative Syndrome Scale (PANSS) and the Wisconsin Card Sorting Test (WCST), showed no association between rs1625579 and schizophrenia was observed in the Belarusian population. Allele and genotype frequencies were specifically investigated in indigenous Belarusian males. While rs1625579 was not linked to schizophrenia, significant interactions between genotype and symptom severity were identified in schizophrenia patients. However, WCST parameters showed no correlation with the rs1625579 polymorphism.

We found a high significance in miR-103a, where there was a downregulation in schizophrenia patients (median: 17.42) compared to the control group (median: 15.16) (*p* = 0.000 HS). Shi, Du^47^ suggested that the miR-103 might be a key player in reflecting the schizophrenia illnesses status and may serve as a candidate biomarker for the diagnosis of schizophrenia. They also found that miR-103 was highly consistent between sporadic schizophrenia and family schizophrenia patients.

Phase II of the North American Prodrome Longitudinal Study (NAPLS-2) assessed symptoms prospectively in participants at clinical high risk (CHR) and in controls over 5 years. A subset of patients consented to blood collections, from which leukocytes were harvested for miRNA sequencing. Although no single miRNA was identified as significantly different across the groups, subjects who progressed to psychosis differed significantly in the combined levels of five miRNAs: one upregulated miRNA (miR-103a-3p)^89,90^.

A later study utilizing the same NAPLS-2 miRNA-seq data set examined the relations between leukocyte miRNAs and gray matter reduction in the superior frontal cortex. Although cortical thinning is a normal feature of adolescent brain development, the rate of cortical thinning is accelerated in subjects at CHR who progress to psychosis. A set of nine miRNAs was used to develop a classifier function to predict the rate of cortical thinning in the NAPLS-2 data set one of them was miR-103a-3p. Notably, although the same miRNA-seq data were used in both studies, only miR-103a-3p appeared in both the miRNA classifier set associated with conversion to psychosis and the classifier set associated with cortical thinning. Neither study found that peripheral miRNA levels are sufficient to predict conversion status, even among individuals at CHR, but together, they suggest that miRNA data may be useful in predicting conversion to psychosis and structural phenotypes associated with that conversion^89,91^.

We found that miR-34a was correlated with the Positive and Negative Syndrome Scale (PANSS), particularly with the general psychopathology scales in schizophrenic patients, in agreement with^92^. Its correlation with the PANSS subscales suggests miR-34a’s involvement in pathways related to psychotic features and stress responses. Additionally, miR-34a could potentially serve as a biomarker for schizophrenia, as its levels may reflect the severity of symptoms. Higher miR-34a levels could be predictive of more severe positive symptoms or general psychopathology, offering insights into treatment response.

Our findings suggest that miR-346 expression does not exhibit significant correlations with cognitive and symptom severity measures, including PANSS, TMT-B, WAIS-II, and WCST. However, a significant inverse correlation was observed between miR-346 expression and the number of trials required to complete the first category (*r* = – 0.398, *p* = 0.01, BH-adjusted), indicating that higher miR-346 levels are associated with improved cognitive flexibility.

A systematic review reported mixed findings regarding miR-346 expression in schizophrenia. Specifically, two studies observed the upregulation of miR-346 in plasma and serum samples, while another noted downregulation in peripheral blood mononuclear cells (PBMCs). These inconsistencies highlight the complexity of miR-346 expression patterns in schizophrenia and suggest the need for further investigation^46^.

Although direct studies linking miR-346 specifically to TMT performance in schizophrenia are limited, the broader role of miRNAs in cognitive processes suggests a potential pathway for future research.

## Conclusion

Schizophrenia is a complex disorder influenced by genetic and environmental factors, with miRNA dysregulation playing a significant role in its pathogenesis. Our study highlights the potential of specific miRNAs, such as miR-137, miR-195, miR-103a, and miR-346, as diagnostic biomarkers and therapeutic targets. These miRNAs demonstrate associations with cognitive function and symptom severity, reflecting their relevance in neurodevelopment and synaptic regulation. The ability to detect miRNAs in accessible biofluids like PBMCs or serum underscores their promise as non-invasive biomarkers, potentially revolutionizing the early diagnosis and monitoring of schizophrenia. Furthermore, correlations between miRNAs and cognitive performance metrics, such as the Trail Making Test and Wisconsin Card Sorting Test, point to their role in understanding cognitive deficits in schizophrenia. Future research should focus on validating these findings across diverse populations and exploring miRNA-based interventions. Integrating miRNA analysis into clinical protocols offers a pathway toward personalized medicine, improving outcomes for individuals with schizophrenia.

## Data Availability

All generated data are presented in the current MS.

## References

[1] Hung, C. C., Lin, C. H. & Lane, H. Y. *Cystine/Glutamate antiporter in schizophrenia: from molecular mechanism to novel biomarker and treatment*. *Int. J. Mol. Sci.*, **22**(18). (2021). 10.3390/ijms22189718 PMC8466274 34575878

[2] Khavari, B. & Cairns, M. J. *Epigenomic dysregulation in schizophrenia: in search of disease etiology and biomarkers*. *Cells*, **9**(8). (2020). 10.3390/cells9081837 PMC7463953 32764320

[3] VosT. et al. Global, regional, and National incidence, prevalence, and years lived with disability for 328 diseases and injuries for 195 countries, 1990–2016: a systematic analysis for the global burden of disease study 2016. *Lancet* **390** (10100), 1211–1259 (2017). 28919117 10.1016/S0140-6736(17)32154-2 PMC5605509

[4] Micale, V. et al. Are the epigenetic changes predictive of therapeutic efficacy for psychiatric disorders? A translational approach towards novel drug targets. *Pharmacol. Ther.* **241**, 108279 (2023). 36103902 10.1016/j.pharmthera.2022.108279

[5] Nagy, N. et al. *Criminality in Psychiatric Patients: Egyptian Study*. (2016).

[6] Zamanpoor, M. Schizophrenia in a genomic era: a review from the pathogenesis, genetic and environmental etiology to diagnosis and treatment insights. *Psychiatr Genet.* **30** (1), 1–9 (2020). 31764709 10.1097/YPG.0000000000000245

[7] Ľupták, M. et al. Novel approaches in schizophrenia-from risk factors and hypotheses to novel drug targets. *World J. Psychiatry*. **11** (7), 277–296 (2021). 34327122 10.5498/wjp.v11.i7.277 PMC8311514

[8] Luvsannyam, E. et al. Neurobiology of schizophrenia: A comprehensive review. *Cureus* **14** (4), e23959 (2022). 35541299 10.7759/cureus.23959 PMC9080788

[9] Kim, M. Understanding the etiology and treatment approaches of schizophrenia: theoretical perspectives and their critique. *Open. J. Psychiatry*. **06**, 253–261 (2016).

[10] McCutcheon, R. A., Reis Marques, T. & Howes, O. D. *Schizophrenia-An Overv. JAMA Psychiatry*, **77**(2): 201–210. (2020). 10.1001/jamapsychiatry.2019.3360 31664453

[11] Setién-Suero, E. et al. Childhood trauma and substance use underlying psychosis: a systematic review. *Eur. J. Psychotraumatology*. **11** (1), 1748342 (2020). 10.1080/20008198.2020.1748342 PMC7191903 32373286

[12] Susser, E. & Patel, V. Psychiatric epidemiology and global mental health: joining forces. *Int. J. Epidemiol.* **43** (2), 287–293 (2014). 24659583 10.1093/ije/dyu053

[13] Fišar, Z. Biological hypotheses, risk factors, and biomarkers of schizophrenia. *Prog. Neuropsychopharmacol. Biol. Psychiatry*. **120**, 110626 (2023). 36055561 10.1016/j.pnpbp.2022.110626

[14] Belbasis, L. et al. Risk factors and peripheral biomarkers for schizophrenia spectrum disorders: an umbrella review of meta-analyses. *Acta Psychiatry. Scand.* **137** (2), 88–97 (2018). 10.1111/acps.12847 29288491

[15] American Psychiatric Association, Diagnostic and statistical manual of mental disorders: DSM-5™, 5th ed. Diagnostic and statistical manual of mental disorders: DSM-5™, 5th ed. Arlington, VA, US: American Psychiatric Publishing, Inc. xliv, 947-xliv, 947. (2013).

[16] Paul, T. et al. Neurostructural changes in schizophrenia and treatment-resistance: a narrative review. *Psychoradiology* **4**, kkae015 (2024). 39399446 10.1093/psyrad/kkae015 PMC11467815

[17] Zhang, H. C. et al. MicroRNA schizophrenia: etiology, biomarkers and therapeutic targets. *Neurosci. Biobehavioral Reviews*. **146**, 105064 (2023). 10.1016/j.neubiorev.2023.105064 36707012

[18] Chen, W. & Qin, C. General hallmarks of MicroRNAs in brain evolution and development. *RNA Biol.* **12** (7), 701–708 (2015). 26000728 10.1080/15476286.2015.1048954 PMC4615839

[19] Chen, Q. et al. *Research Progress on the Correlation between Epigenetics and Schizophrenia*15 (Frontiers in Neuroscience, 2021). 10.3389/fnins.2021.688727 PMC8334178 34366776

[20] Wang, R. et al. Human MicroRNA (miR-20b-5p) modulates Alzheimer’s disease pathways and neuronal function, and a specific polymorphism close to the MIR20B gene influences Alzheimer’s biomarkers. *Mol. Psychiatry*. **27** (2), 1256–1273 (2022). 35087196 10.1038/s41380-021-01351-3 PMC9054681

[21] Nowakowski, T. J. et al. Regulation of cell-type-specific transcriptomes by MicroRNA networks during human brain development. *Nat. Neurosci.* **21** (12), 1784–1792 (2018). 30455455 10.1038/s41593-018-0265-3 PMC6312854

[22] Nguyen, L. S. et al. Role of miR-146a in neural stem cell differentiation and neural lineage determination: relevance for neurodevelopmental disorders. *Mol. Autism*. **9** (1), 38 (2018). 29951184 10.1186/s13229-018-0219-3 PMC6011198

[23] Hu, Z. et al. Temporal dynamics of MiRNAs in human DLPFC and its association with MiRNA dysregulation in schizophrenia. *Translational Psychiatry*. **9** (1), 196 (2019). 31431609 10.1038/s41398-019-0538-y PMC6702224

[24] Shorter, K. R. & Miller, B. H. Epigenetic mechanisms in schizophrenia. *Prog. Biophys. Mol. Biol.* **118** (1), 1–7 (2015). 25958205 10.1016/j.pbiomolbio.2015.04.008 PMC4631256

[25] Moszyńska, A. et al. *SNPs in MicroRNA target sites and their potential role in human disease*. *Open. Biol.*, **7**(4). (2017). 10.1098/rsob.170019 PMC5413909 28381629

[26] Hollins, S. L. et al. Ontogeny of small RNA in the regulation of mammalian brain development. *BMC Genom.* **15** (1), 777 (2014). 10.1186/1471-2164-15-777 PMC4171549 25204312

[27] Perkins, D. O. et al. MicroRNA expression in the prefrontal cortex of individuals with schizophrenia and schizoaffective disorder. *Genome Biol.* **8** (2), R27 (2007). 17326821 10.1186/gb-2007-8-2-r27 PMC1852419

[28] Wojtalik, J. A. et al. A systematic and Meta-analytic review of neural correlates of functional outcome in schizophrenia. *Schizophr Bull.* **43** (6), 1329–1347 (2017). 28204755 10.1093/schbul/sbx008 PMC5737663

[29] Dirnberger, G. et al. Neural correlates of executive dysfunction in schizophrenia: failure to modulate brain activity with task demands. *Neuroreport* **25** (16), 1308–1315 (2014). 25275638 10.1097/WNR.0000000000000264

[30] Gibbons, A., Udawela, M. & Dean, B. Non-Coding RNA as novel players in the pathophysiology of schizophrenia. *Non-Coding RNA*. **4** (2), 11 (2018). 29657307 10.3390/ncrna4020011 PMC6027250

[31] Beveridge, N. J. et al. Schizophrenia is associated with an increase in cortical MicroRNA biogenesis. *Mol. Psychiatry*. **15** (12), 1176–1189 (2010). 19721432 10.1038/mp.2009.84 PMC2990188

[32] Miller, B. H. et al. MicroRNA-132 dysregulation in schizophrenia has implications for both neurodevelopment and adult brain function. *Proc. Natl. Acad. Sci. U S A*. **109** (8), 3125–3130 (2012). 22315408 10.1073/pnas.1113793109 PMC3286960

[33] He, K. et al. MiRNAs of peripheral blood as the biomarker of schizophrenia. *Hereditas* **155**, 9 (2018). 28860957 10.1186/s41065-017-0044-2 PMC5575894

[34] Vallès, A. et al. MicroRNA-137 regulates a glucocorticoid receptor-dependent signalling network: implications for the etiology of schizophrenia. *J. Psychiatry Neurosci.* **39** (5), 312–320 (2014). 24866554 10.1503/jpn.130269 PMC4160360

[35] Steudle, F. et al. A novel de Novo variant of GABRA1 causes increased sensitivity for GABA in vitro. *Sci. Rep.* **10** (1), 2379 (2020). 32047208 10.1038/s41598-020-59323-6 PMC7012862

[36] Gibbons, A., Udawela, M. & Dean, B. *Non-Coding RNA as novel players in the pathophysiology of schizophrenia*. *Noncoding RNA*, **4**(2). (2018). 10.3390/ncrna4020011 PMC6027250 29657307

[37] Kuswanto, C. N. et al. The impact of genome wide supported microRNA-137 (MIR137) risk variants on frontal and striatal white matter integrity, neurocognitive functioning, and negative symptoms in schizophrenia. *Am. J. Med. Genet. B Neuropsychiatr Genet.* **168b** (5), 317–326 (2015). 25921703 10.1002/ajmg.b.32314

[38] Ma, G. et al. *Association of a miRNA-137 polymorphism with schizophrenia in a Southern Chinese Han population.* Biomed Res Int, 2014: p. 751267. (2014). 10.1155/2014/751267 PMC4163463 25250332

[39] Li, Z. et al. MiR-195 inhibits the proliferation of human cervical cancer cells by directly targeting Cyclin D1. *Tumour Biol.* **37** (5), 6457–6463 (2016). 26631043 10.1007/s13277-015-4540-6

[40] Sun, X. Y. et al. Aberrant MicroRNA expression in peripheral plasma and mononuclear cells as specific blood-based biomarkers in schizophrenia patients. *J. Clin. Neurosci.* **22** (3), 570–574 (2015). 25487174 10.1016/j.jocn.2014.08.018

[41] Guo, A. Y. et al. A novel MicroRNA and transcription factor mediated regulatory network in schizophrenia. *BMC Syst. Biol.* **4** (1), 10 (2010). 20156358 10.1186/1752-0509-4-10 PMC2834616

[42] Pan, S. et al. The microRNA-195 - BDNF pathway and cognitive deficits in schizophrenia patients with minimal antipsychotic medication exposure. *Translational Psychiatry*. **11** (1), 117 (2021). 33558459 10.1038/s41398-021-01240-x PMC7870897

[43] Lu, B., Nagappan, G. & Lu, Y. BDNF and synaptic plasticity, cognitive function, and dysfunction. *Handb. Exp. Pharmacol.* **220**, 223–250 (2014). 24668475 10.1007/978-3-642-45106-5_9

[44] Zaki, M. B. et al. The potential role of MiRNAs in the pathogenesis of schizophrenia – A focus on signaling pathways interplay. *Pathol. - Res. Pract.* **254**, 155102 (2024). 38211386 10.1016/j.prp.2024.155102

[45] Yang, Y. et al. Brain-derived neurotrophic factor is associated with cognitive impairments in first-episode and chronic schizophrenia. *Psychiatry Res.* **273**, 528–536 (2019). 30710808 10.1016/j.psychres.2019.01.051

[46] Grosu, Ș. A., et al., *Blood-Based MicroRNAs in psychotic Disorders-A systematic review*. *Biomedicines*, **11**(9). (2023). 10.3390/biomedicines11092536 PMC10525934 37760977

[47] Shi, W. et al. Aberrant expression of serum MiRNAs in schizophrenia. *J. Psychiatr Res.* **46** (2), 198–204 (2012). 22094284 10.1016/j.jpsychires.2011.09.010

[48] Yin, J. et al. miR-137: a new player in schizophrenia. *Int. J. Mol. Sci.* **15** (2), 3262–3271 (2014). 24566148 10.3390/ijms15023262 PMC3958910

[49] Mu, C., Dang, X. & Luo, X. J. Mendelian randomization reveals the causal links between MicroRNA and schizophrenia. *J. Psychiatr Res.* **163**, 372–377 (2023). 37267734 10.1016/j.jpsychires.2023.05.071

[50] Segaran, R. C. et al. Neuronal Development-Related MiRNAs as biomarkers for Alzheimer’s disease, depression, schizophrenia and ionizing radiation exposure. *Curr. Med. Chem.* **28** (1), 19–52 (2021). 31965936 10.2174/0929867327666200121122910

[51] He, K. et al. Identification of serum MicroRNAs as diagnostic biomarkers for schizophrenia. *Hereditas* **156**, 23 (2019). 31297041 10.1186/s41065-019-0099-3 PMC6598381

[52] Jufe, G. S. [Schizophrenia according to DSM-5]. *Vertex* **25** (113), 36–42 (2014). 24887368

[53] First, M. B. Diagnostic and statistical manual of mental disorders, 5th edition, and clinical utility. *J. Nerv. Ment Dis.* **201** (9), 727–729 (2013). 23995026 10.1097/NMD.0b013e3182a2168a

[54] Faul, F. et al. G*Power 3: a flexible statistical power analysis program for the social, behavioral, and biomedical sciences. *Behav. Res. Methods*. **39** (2), 175–191 (2007). 17695343 10.3758/bf03193146

[55] Laere, E., Tee, S. F. & Tang, P. Y. Assessment of cognition in schizophrenia using trail making test: A Meta-Analysis. *Psychiatry Investig*. **15** (10), 945–955 (2018). 30223641 10.30773/pi.2018.07.22 PMC6212701

[56] Fujino, H. et al. Performance on the Wechsler adult intelligence Scale-III in Japanese patients with schizophrenia. *Psychiatry Clin. Neurosci.* **68** (7), 534–541 (2014). 24447376 10.1111/pcn.12165

[57] PEBL. The PEBL Project. ; (2019). Available from: https://pebl.sourceforge.net

[58] Mueller, S. T. & Piper, B. J. The psychology experiment Building Language (PEBL) and PEBL test battery. *J. Neurosci. Methods*. **222**, 250–259 (2014). 24269254 10.1016/j.jneumeth.2013.10.024 PMC3897935

[59] Chen, Q. et al. Research progress on the correlation between epigenetics and schizophrenia. *Front. Neurosci.* **15**, 688727 (2021). 34366776 10.3389/fnins.2021.688727 PMC8334178

[60] Legge, S. E. et al. Genetic architecture of schizophrenia: a review of major advancements. *Psychol. Med.* **51** (13), 2168–2177 (2021). 33550997 10.1017/S0033291720005334

[61] Tsujimura, K., Shiohama, T. & Takahashi, E. MicroRNA biology on brain development and neuroimaging approach. *Brain Sci.* **12** (10), 1366 (2022). 36291300 10.3390/brainsci12101366 PMC9599180

[62] Shboul, M. et al. Plasma MiRNAs as potential biomarkers for schizophrenia in a Jordanian cohort. *Noncoding RNA Res.* **9** (2), 350–358 (2024). 38511065 10.1016/j.ncrna.2024.01.018 PMC10950580

[63] Moreau, M. P. et al. Altered MicroRNA expression profiles in postmortem brain samples from individuals with schizophrenia and bipolar disorder. *Biol. Psychiatry*. **69** (2), 188–193 (2011). 21183010 10.1016/j.biopsych.2010.09.039 PMC3038345

[64] Hollins, S. L. et al. Alteration of imprinted Dlk1-Dio3 MiRNA cluster expression in the entorhinal cortex induced by maternal immune activation and adolescent cannabinoid exposure. *Translational Psychiatry*. **4** (9), e452–e452 (2014). 25268256 10.1038/tp.2014.99 PMC4203021

[65] Du, Y. et al. Genome-Wide, integrative analysis implicates Exosome-Derived MicroRNA dysregulation in schizophrenia. *Schizophr. Bull.* **45** (6), 1257–1266 (2019). 30770930 10.1093/schbul/sby191 PMC6811837

[66] Du, Y. et al. Metabolomic identification of Exosome-Derived biomarkers for schizophrenia: A large multicenter study. *Schizophr. Bull.* **47** (3), 615–623 (2020). 10.1093/schbul/sbaa166 PMC8084447 33159208

[67] Zhong, X. L. et al. Unlocking the therapeutic potential of exosomes derived from nasal olfactory mucosal mesenchymal stem cells: restoring synaptic plasticity, neurogenesis, and neuroinflammation in schizophrenia. *Schizophr. Bull.* **50** (3), 600–614 (2023). 10.1093/schbul/sbad172 PMC11059802 38086528

[68] Riecher-Rössler, A., Butler, S. & Kulkarni, J. Sex and gender differences in schizophrenic psychoses—a critical review. *Arch. Women Ment. Health*. **21** (6), 627–648 (2018). 10.1007/s00737-018-0847-9 29766281

[69] Novick, D. et al. Regional differences in treatment response and three year course of schizophrenia across the world. *J. Psychiatr. Res.* **46** (7), 856–864 (2012). 22575332 10.1016/j.jpsychires.2012.03.017

[70] Giordano, G. M. et al. *Gender differences in clinical and psychosocial features among persons with schizophrenia: A Mini review*. *Front. Psychiatry*, 12. (2021). 10.3389/fpsyt.2021.789179 PMC8727372 35002807

[71] Wei, H. et al. Detection of Circulating MiRNA levels in schizophrenia. *Am. J. Psychiatry*. **172** (11), 1141–1147 (2015). 26183697 10.1176/appi.ajp.2015.14030273

[72] Coustals, N. et al. Chronic smoking and cognition in patients with schizophrenia: A meta-analysis. *Schizophr. Res.* **222**, 113–121 (2020). 32507373 10.1016/j.schres.2020.03.071

[73] Castle, D., Baker, A. L. & Bonevski, B. Editorial: smoking and schizophrenia. *Front. Psychiatry*. **10**, 738 (2019). 31681044 10.3389/fpsyt.2019.00738 PMC6812412

[74] Kelly, C. & McCreadie, R. G. Smoking habits, current symptoms, and premorbid characteristics of schizophrenic patients in Nithsdale, Scotland. *Am. J. Psychiatry*. **156** (11), 1751–1757 (1999). 10553739 10.1176/ajp.156.11.1751

[75] Dickerson, F. et al. Cigarette smoking by patients with serious mental illness, 1999–2016: an increasing disparity. *Psychiatr Serv.* **69** (2), 147–153 (2018). 28945183 10.1176/appi.ps.201700118

[76] de Leon, J. & Diaz, F. J. A meta-analysis of worldwide studies demonstrates an association between schizophrenia and tobacco smoking behaviors. *Schizophr. Res.* **76** (2), 135–157 (2005). 15949648 10.1016/j.schres.2005.02.010

[77] Abassi, B. et al. Smoking in patients with schizophrenia: no smoking without fire. *Eur. Psychiatry*. **65** (S1), S786–S786 (2022).

[78] Hartz, S. M. et al. Genetic correlation between smoking behaviors and schizophrenia. *Schizophr. Res.* **194**, 86–90 (2018). 28285025 10.1016/j.schres.2017.02.022 PMC5811408

[79] Isuru, A. & Rajasuriya, M. Tobacco smoking and schizophrenia: re-examining the evidence. *BJPsych Adv.* **25** (6), 363–372 (2019).

[80] He, J. F. et al. Biogenesis of MiRNA-195 and its role in biogenesis, the cell cycle, and apoptosis. *J. Biochem. Mol. Toxicol.* **25** (6), 404–408 (2011). 22190509 10.1002/jbt.20396

[81] Huang, X. et al. MicroRNA-195 predicts olanzapine response in drug-free patients with schizophrenia: A prospective cohort study. *J. Psychopharmacol.* **35** (1), 23–30 (2021). 33274684 10.1177/0269881120959617

[82] Yin, J. et al. miR-137: A new player in schizophrenia. *Int. J. Mol. Sci.* **15**, 3262–3271 (2014). 24566148 10.3390/ijms15023262 PMC3958910

[83] Ou, M. et al. *Association between miR-137 Polymorphism and Risk of Schizophrenia: a meta-analysis* (GMR, 2016). 15 3. 10.4238/gmr.15038703 27706734

[84] Wu, S. et al. MicroRNA-137 inhibits EFNB2 expression affected by a genetic variant and is expressed aberrantly in peripheral blood of schizophrenia patients. *EBioMedicine* **12**, 133–142 (2016). 27650867 10.1016/j.ebiom.2016.09.012 PMC5078603

[85] Chen, B. Y. et al. Neurodevelopment regulators miR-137 and miR-34 family as biomarkers for early and adult onset schizophrenia. *NPJ Schizophr*. **7** (1), 35 (2021). 34226568 10.1038/s41537-021-00164-1 PMC8257739

[86] Ma, J. et al. Identification of miR-22-3p, miR-92a-3p, and miR-137 in peripheral blood as biomarker for schizophrenia. *Psychiatry Res.* **265**, 70–76 (2018). 29684772 10.1016/j.psychres.2018.03.080

[87] He, E. et al. MIR137 schizophrenia-associated locus controls synaptic function by regulating synaptogenesis, synapse maturation and synaptic transmission. *Hum. Mol. Genet.* **27**, 1879–1891 (2018). 29635364 10.1093/hmg/ddy089 PMC5961183

[88] Kandratsenka, H. et al. *Association of MIR137 with Symptom Severity and Cognitive Functioning in Belarusian Schizophrenia Patients*9 (Frontiers in Psychiatry, 2018). 10.3389/fpsyt.2018.00295 PMC6041593 30026708

[89] Thomas, K. T. & Zakharenko, S. S. MicroRNAs in the onset of schizophrenia. *Cells* **10** (10), 2679 (2021). 34685659 10.3390/cells10102679 PMC8534348

[90] Jeffries, C. D. et al. Insights into psychosis risk from leukocyte MicroRNA expression. *Transl Psychiatry*. **6** (12), e981 (2016). 27959328 10.1038/tp.2016.148 PMC5290334

[91] Zheutlin, A. B. et al. The role of MicroRNA expression in cortical development during conversion to psychosis. *Neuropsychopharmacology* **42** (11), 2188–2195 (2017). 28186095 10.1038/npp.2017.34 PMC5603810

[92] Sun, X. et al. A preliminary analysis of MicroRNA as potential clinical biomarker for schizophrenia. *Am. J. Med. Genet. Part. B: Neuropsychiatric Genet.* **168** (3), 170–178 (2015). 10.1002/ajmg.b.32292 25656957

